# Entanglement monogamy in three qutrit systems

**DOI:** 10.1038/s41598-017-02066-8

**Published:** 2017-05-16

**Authors:** Qiting Li, Jianlian Cui, Shuhao Wang, Gui-Lu Long

**Affiliations:** 1 Department of Mathematical Sciences, Tsinghua University, Beijing, 100084 P. R. China; 2 State Key Laboratory of Low-Dimensional Quantum Physics and Department of Physics, Tsinghua University, Beijing, 100084 P. R. China; 3 Tsinghua National Laboratory for Information Science and Technology, Beijing, 100084 P. R. China; 4 Collaborative Innovation Center of Quantum Matter, Beijing, 100084 P. R. China

## Abstract

By introducing an arbitrary-dimensional multipartite entanglement measure, which is defined in terms of the reduced density matrices corresponding to all possible two partitions of the entire system, we prove that multipartite entanglement cannot be freely shared among the parties in both *n*-qubit systems and three-qutrit systems. Furthermore, our result implies that the satisfaction of the entanglement monogamy is related to the number of particles in the quantum system. As an application of three-qutrit monogamy inequality, we give a condition for the separability of a class of two-qutrit mixed states in a 3 ⊗ 3 system.

## Introduction

Quantum entanglement is an essential feature of quantum mechanics, which distinguishes the quantum from the classical world. Because of entanglement, different quantum systems can affect each other, even if there is no classical connection between the multiple quantum systems. So quantum entanglement can be used to perform a number of tasks which can not be completed in the classical mechanical system. Quantification of quantum entanglement plays an important role in quantum information processing and quantum computation^1–5^. The mathematical study of entanglement has become a very active field and has led to many operational and information theoretic insights.

Entanglement is monogamous, which was first discovered by tangle for three qubit systems in the seminal paper of Coffiman, Kundu and Wootters^6^. It describes the constraint on distributed entanglement among many parties. It is also a key ingredient in quantum cryptography security^7, 8^, statistical mechanics^9^, the foundations of quantum mechanics^10^ and black-hole physics^11^. In addition to having a wide range of practical applications, monogamy has also profound theoretical significance, allowing simplified proofs of no-broadcasting bounds and constraints for qubit multitap channel capacities^12^.

The author stated in ref. 12 that the monogamy inequality in the condensed matter physics gives rise to the frustration effects observed in, e.g., Heisenberg antiferromagnets. The perfect ground state for an antiferromagnet would in fact consist of singlets between all interacting spins. However, as a particle can only share one unit of entanglement with all its neighbors, it will try to spread its entanglement in an optimal way with all its neighbors leading to a strongly correlated ground state. Such qualitative statements have been turned into quantitative ones in *n*-qubit systems through the square of the concurrence^12^, the square of the entanglement of formation^13^ and the square of convex-roof extended negativity^14^, respectively.

Suppose that *E* is an entanglement measure for the multipartite system ${ {\mathcal H} }_{1}\otimes { {\mathcal H} }_{2}\otimes \cdots \otimes { {\mathcal H} }_{n}$. Monogamous relation expressed in terms of inequalities can be represented as

$${E}_{1|2\cdots n}\geqslant {E}_{1|2}+{E}_{1|3}+\cdots +{E}_{\mathrm{1|}n},$$

where ${E}_{\mathrm{1|2}\cdots n}$ denotes the bipartite quantum entanglement across the bipartition ${ {\mathcal H} }_{1}|{ {\mathcal H} }_{2}\cdots { {\mathcal H} }_{n}$, *E*_1|*j*_ denotes the bipartite quantum entanglement between ${ {\mathcal H} }_{1}$ and ${ {\mathcal H} }_{j}$.

In ref. 15, we proposed an entanglement measure for arbitrary dimensional multipartite systems based on the weighed average of the square of fidelity and proved that it satisfied monogamous relation for three qubit systems. In this paper, we will first generalize the monogamy inequality to *n*-qubit systems.

The authors in ref. 16 by an anti-example pointed out that the monogamy inequality characterized by the square of concurrence cannot be generalized to a quantum system apart from qubits. This raises a fundamental physical question: does there exist the monogamy in the higher dimensional systems? In this paper, for three-qutrit systems, we obtain an analytic expression of our measure, and furthermore, answer positively the question.

Our results clearly elucidates the restriction on the sharing of entanglement among both *n*-qubit systems and three qutrit systems. In addition, we obtain that the satisfaction of the entanglement monogamy characterized by an entanglement measure is generally related to the number of particles in the quantum system.

## Results

Let ${ {\mathcal H} }^{{d}_{i}}$ be a Hilbert space with dimension *d*_*i*_, $i=1,2,\ldots ,n$. For an *n*-qudit pure state |*ψ*〉 in the *n*-partite quantum system ${\mathcal H} ={ {\mathcal H} }^{{d}_{1}}\otimes { {\mathcal H} }^{{d}_{2}}\otimes \cdots \otimes { {\mathcal H} }^{{d}_{n}}$, define

1 $${ {\mathcal E} }^{M}(|\psi \rangle )=\mathop{{\rm{\min }}}\limits_{{{\mathscr{A}}}_{2}}\,{\tilde{d}}^{n-2}[{(tr\sqrt{{\rho }_{{A}_{1}}})}^{2}-1],$$

where ${\rho }_{{A}_{1}}$ is the reduced density matrix of |*ψ*〉 〈*ψ*| on subsystem ${ {\mathcal H} }_{{A}_{1}}$; the minimum $\mathop{{\rm{\min }}}\limits_{{{\mathscr{A}}}_{2}}$ is taken over all possible 2-partitions ${{\mathscr{A}}}_{2}={A}_{1}|{A}_{2}$ of the system ${\mathcal H}$ and $\tilde{d}=\frac{{\sum }_{i=1}^{n}\,{d}_{i}}{n}$. For an *n*-qudit mixed state *ρ* in the *n*-partite quantum system ${\mathcal H}$, we define

$${ {\mathcal E} }^{M}(\rho )=\mathop{{\rm{\inf }}}\limits_{\{{p}_{i},|{\psi }_{i}\rangle \}}\sum _{i}\,{p}_{i}{ {\mathcal E} }^{M}(|{\psi }_{i}\rangle ),$$

where the infimum is taken over all possible pure state decompositions $\rho ={\sum }_{i}\,{p}_{i}|{\psi }_{i}\rangle \langle {\psi }_{i}|$. Analogously to ref. 15, we can check that ${ {\mathcal E} }^{M}$ is an entanglement measure. We can also verify that ${ {\mathcal E} }^{M}$ satisfies the convexity (monotonicity under discarding information)^17^ for any states:

2 $${ {\mathcal E} }^{M}(\sum _{i}\,{p}_{i}{\rho }_{i})\leqslant \sum _{i}\,{p}_{i}{ {\mathcal E} }^{M}({\rho }_{i}).$$

A similar discussion just as in ref. 15 implies that ${ {\mathcal E} }^{M}$ satisfies the monogamous relation for three-qubit quantum systems. The following theorem generalizes this result to the case of *n*-qubit systems.

**Theorem 1**. For a *n*-qubit system, ${ {\mathcal E} }^{M}$ satisfies the monogamy inequality, i.e.,

$${ {\mathcal E} }_{\mathrm{1|2}\cdots n}^{M}\geqslant { {\mathcal E} }_{\mathrm{1|2}}^{M}+{ {\mathcal E} }_{\mathrm{1|3}}^{M}+\cdots +{ {\mathcal E} }_{\mathrm{1|}n}^{M}.$$

The proof of this theorem can be found in the Supplemental Material.

For *n*-qudit pure state |*ψ*〉 in the system ${\mathcal H} ={ {\mathcal H} }^{{d}_{1}}\otimes { {\mathcal H} }^{{d}_{2}}\otimes \cdots \otimes { {\mathcal H} }^{{d}_{n}}$, let

3 $${\mathscr{C}}(|\psi \rangle )=\mathop{{\rm{\min }}}\limits_{{{\mathscr{A}}}_{2}}\sqrt{2(1-tr{\rho }_{{A}_{1}}^{2})},$$

where the minimum $\mathop{{\rm{\min }}}\limits_{{{\mathscr{A}}}_{2}}$ is taken over all possible 2-partitions ${{\mathscr{A}}}_{2}={A}_{1}|{A}_{2}$ of the system ${\mathcal H}$. Clearly ${\mathscr{C}}$ is an entanglement measure.

Consider the state $|GHZ\rangle =\frac{1}{\sqrt{3}}(|100\rangle +|010\rangle +|001\rangle )$, we find that ${{\mathscr{C}}}_{AB}(|GHZ\rangle )={{\mathscr{C}}}_{AC}(|GHZ\rangle )=\frac{2}{3}$ and ${{\mathscr{C}}}_{A|BC}(|GHZ\rangle )=\frac{2\sqrt{2}}{3}$ (see ref. 6). Hence ${{\mathscr{C}}}_{AB}+{{\mathscr{C}}}_{AC} > {{\mathscr{C}}}_{A|BC}$, which violates the monogamy inequality.

Now we add a coefficient which is related to the particle number of systems in Eq. (3), and let

4 $${{\mathscr{C}}}^{N}(|\psi \rangle )=\mathop{{\rm{\min }}}\limits_{{{\mathscr{A}}}_{2}}\,{\tilde{d}}^{n-2}\sqrt{2(1-tr{\rho }_{{A}_{1}}^{2})},$$

where $\tilde{d}=\frac{{\sum }_{i=1}^{n}\,{d}_{i}}{n}$. Observe that, if *ρ* is a rank two state, then $\sqrt{2(1-tr{\rho }^{2})}={(tr\sqrt{\rho })}^{2}-1$. Thus, the following result follows immediately from Theorem 1.

**Corollary 1**. For an 3-qubit system, ${{\mathscr{C}}}^{N}$ satisfies the monogamy inequality.

The above discussion implies that the concurrence itself does not satisfy monogamous relation. This, together with Corollary 1, shows that the satisfaction of entanglement monogamy characterized by an entanglement measure is generally related to the number of particles of the system.

Next we discuss the entanglement monogamy in three qutrit systems. Until now, no true entanglement measure has been proven to be monogamous for three-dimensional tripartite systems. Taking the square of concurrence as an example, an explicit counterexample showing the violation of the monogamy inequality in three-dimensional quantum systems is as follows^16^,

$$\begin{matrix}|{\rm{\Psi }}\rangle  & = & \frac{1}{\sqrt{6}}({|0\rangle }_{A}{|1\rangle }_{B}{|2\rangle }_{c}-{|0\rangle }_{A}{|2\rangle }_{B}{|1\rangle }_{C}+{|1\rangle }_{A}{|2\rangle }_{B}{|0\rangle }_{C}\\  &  & -\,{|1\rangle }_{A}{|0\rangle }_{B}{|2\rangle }_{C}+{|2\rangle }_{A}{|0\rangle }_{B}{|1\rangle }_{C}-{|2\rangle }_{A}{|1\rangle }_{B}{|0\rangle }_{C}).\end{matrix}$$

Clearly, the density matrix ${\rho }_{A}={{\rm{tr}}}_{BC}|{\rm{\Psi }}\rangle \langle {\rm{\Psi }}|$ has the spectrum $\{\frac{1}{3},\frac{1}{3},\frac{1}{3}\}$. For an arbitrary pure state |Φ〉_*AB*_, a discussion just as in ref. 16 implies that the reduced density matrix ${\rho }_{A}={{\rm{tr}}}_{B}{|{\rm{\Phi }}\rangle }_{AB}\langle {\rm{\Phi }}|$ has the same spectrum $\{\frac{1}{2},\frac{1}{2},0\}$ (see also ref. 18).

For the square of the concurrence, it follows that ${{\mathscr{C}}}_{A|BC}^{2}=\frac{4}{3}$ and ${{\mathscr{C}}}_{AB}^{2}={{\mathscr{C}}}_{AC}^{2}=1$, hence, ${{\mathscr{C}}}_{AB}^{2}+{{\mathscr{C}}}_{AC}^{2}=2 > \frac{4}{3}={{\mathscr{C}}}_{A|BC}^{2}$, which means that the square of concurrence does not work for monogamy inequality on a three-qutrit system. Using the entanglement measure ${ {\mathcal E} }^{M}$, it can be calculated that ${ {\mathcal E} }_{AB}^{M}={ {\mathcal E} }_{AC}^{M}\leqslant 1$ and ${ {\mathcal E} }_{A|BC}^{M}=6$. Therefore, ${ {\mathcal E} }_{AB}^{M}+{ {\mathcal E} }_{AC}^{M} < { {\mathcal E} }_{A|BC}^{M}$. More generally, we will prove that the measure ${ {\mathcal E} }^{M}$ satisfies the monogamy inequality in a three-qutrit system. As a first step toward proving this inequality, we will now derive a computable formula for ${ {\mathcal E} }^{M}$.

**Lemma 1**. Let ${\alpha }_{i}\in {{\mathbb{C}}}^{m}(m\leqslant 3)$ be *m*-dimensional complex column vectors (*i* = 1, 2, 3) satisfying ${\sum }_{i=1}^{3}\,{\Vert {\alpha }_{i}\Vert }^{2}=1$. Let $\rho ={(\langle {\alpha }_{j},{\alpha }_{i}\rangle )}_{ij}$ be a 3 × 3 matrix with $1\leqslant i$, $j\leqslant 3$. Here 〈·, ·〉 is the inner product in ${{\mathbb{C}}}^{m}$. Denote by *det*(*ρ*) the determinant of *ρ*. Then

$$tr\sqrt{\rho }=\frac{\sqrt{2+y}+\sqrt{2-y+2\sqrt{{y}^{2}-4+16{\rm{\Delta }}}}}{2},$$

where

$$\begin{matrix}y & = & 2\sqrt{-\frac{p}{3}}\,\cos \,\theta -\frac{2}{3}\,{\rm{and}}\\ {\rm{\Delta }} & = & \sum _{1\leqslant i < j\leqslant 3}{\Vert {\alpha }_{i}\Vert }^{2}{\Vert {\alpha }_{j}\Vert }^{2}-\sum _{1\leqslant i < j\leqslant 3}{|\langle {\alpha }_{i},{\alpha }_{j}\rangle |}^{2}\end{matrix}$$

with $$p=16({\rm{\Delta }}-\tfrac{1}{3})\le 0$$, $$\theta =\tfrac{1}{3}\,\arccos \,(-\tfrac{q}{2r})$$, $$q=\tfrac{64}{3}{\rm{\Delta }}-\tfrac{128}{27}-64det(\rho )$$ and $$r=\sqrt{-{(\tfrac{p}{3})}^{3}}$$.

The proof of this lemma will be given in the Supplemental Material.

**Theorem 2**. For a three-qutrit system ${ {\mathcal H} }_{A}\otimes { {\mathcal H} }_{B}\otimes { {\mathcal H} }_{C}$, ${ {\mathcal E} }^{M}$ satisfies the monogamy inequality, i.e.,

$${ {\mathcal E} }_{A|BC}^{M}\geqslant { {\mathcal E} }_{AB}^{M}+{ {\mathcal E} }_{AC}^{M}.$$

See Methods for the proof of this theorem.

## Discussions

The monogamy of entanglement characterized by the entanglement measure describes quantitatively the entanglement between quantum systems. Choosing the proper entanglement measure helps to reveal the nature of entanglement. The more system information reflected by an entanglement measure, the better it can describe the entanglement of the system. Through giving an entanglement measure which is related to the number of particles of the system, we prove that multipartite entanglements cannot be freely shared among the parties in both *n*-qubit systems and three-qutrit systems. Corollary 1 and the discussion perior to Corollary 1 imply that the satisfaction of entanglement monogamy characterized by an entanglement measure is generally connected with the number of particles of the system.

For the state |Ψ〉 given before Lemma 1 in a three qutrit system ${ {\mathcal H} }_{A}\otimes { {\mathcal H} }_{B}\otimes { {\mathcal H} }_{C}$, one can compute ${{\mathscr{C}}}_{A|BC}^{N}={{\mathscr{C}}}^{N}(|{\rm{\Psi }}\rangle )=2\sqrt{3}$, ${{\mathscr{C}}}_{AB}^{N}={{\mathscr{C}}}^{N}({\rho }_{AB}(|{\rm{\Psi }}\rangle ))=1$ and ${{\mathscr{C}}}_{AC}^{N}={{\mathscr{C}}}^{N}({\rho }_{AC}(|{\rm{\Psi }}\rangle ))=1$, so ${{\mathscr{C}}}_{AB}^{N}+{{\mathscr{C}}}_{AC}^{N} < {{\mathscr{C}}}_{A|BC}^{N}$, that is, the monogamy inequality holds, where ${{\mathscr{C}}}^{N}$ is defined in Eq. (4). More generally, we conjecture that the entanglement measure ${{\mathscr{C}}}^{N}$ satisfies the monogamy inequality in three qutrit systems. As a subsequent work, we will continue to discuss it.

In addition, the entanglement monogamy inequality gives an upper bound for the entanglement degree of two-qutrit mixed states, for which the general separability criteria and computable entanglement measures remain still open. In the Supplemental Material, by such an upper bound, a condition is given for the separability of a class of two-qutrit mixed states in a 3 ⊗ 3 system.

## Methods

### Proof of Theorem 2

Let |*ϕ*〉_*ABC*_ be a pure state in the three-qutrit system ${ {\mathcal H} }_{A}\otimes { {\mathcal H} }_{B}\otimes { {\mathcal H} }_{C}$, then

5 $${|\varphi \rangle }_{ABC}=\sum _{0\leqslant i,j,k\leqslant 2}\,{\beta }_{ijk}{|i\rangle }_{A}{|j\rangle }_{B}{|k\rangle }_{C},$$

where ${\sum }_{0\leqslant i,j,k\leqslant 2}\,{|{\beta }_{ijk}|}^{2}=1$ and $\{{|i\rangle }_{A}|i=0,1,2\}$, $\{{|j\rangle }_{B}|j=0,1,2\}$ and $\{{|k\rangle }_{C}|k=0,1,2\}$ are the orthonormal bases of the qutrit system ${ {\mathcal H} }_{A}$, ${ {\mathcal H} }_{B}$ and ${ {\mathcal H} }_{C}$, respectively. Let

$${h}_{i}={({\beta }_{i00},{\beta }_{i01},{\beta }_{i02},{\beta }_{i10},{\beta }_{i11},{\beta }_{i12},{\beta }_{i20},{\beta }_{i21},{\beta }_{i22})}^{T},\,i=0,1,2.$$

Here, *a* ^*T*^ denotes the transposition of the vector *a*. One can obtain that

$$\begin{matrix}{\rho }_{A}({|\varphi \rangle }_{ABC}) & = & {\rho }_{A00}{|0\rangle }_{A}\langle 0|+{\rho }_{A01}{|0\rangle }_{A}\langle 1|+{\rho }_{A02}{|0\rangle }_{A}\langle 2|+{\rho }_{A10}{|1\rangle }_{A}\langle 0|\\  &  & +\,{\rho }_{A11}{|1\rangle }_{A}\langle 1|+{\rho }_{A12}{|1\rangle }_{A}\langle 2|+{\rho }_{A20}{|2\rangle }_{A}\langle 0|\\  &  & +\,{\rho }_{A21}{|2\rangle }_{A}\langle 1|+{\rho }_{A22}{|2\rangle }_{A}\langle 2|,\end{matrix}$$

where $${\rho }_{Aij}=\langle {h}_{i},{h}_{j}\rangle $$, $$0\leqslant i$$, $$j\leqslant 2$$.

Using Lemma 1, we calculate the entanglement between the particle *A* and the particles *BC*,

$$\begin{matrix}{ {\mathcal E} }_{A|BC}^{M} & = & 3[{(tr\sqrt{{\rho }_{A}({|\psi \rangle }_{ABC})})}^{2}-1]\\  & = & \frac{3}{2}\cdot \frac{{(\sqrt{2+{y}_{A}}+\sqrt{2-{y}_{A}+2\sqrt{{y}_{A}^{2}-4(1-4{{\rm{\Delta }}}_{A})}})}^{2}-4}{2},\end{matrix}$$

where

$$\begin{matrix}{y}_{A} & = & 2\sqrt{-\frac{{p}_{A}}{3}}\,\cos \,{\theta }_{A}-\frac{2}{3}\,{\rm{and}}\\ {{\rm{\Delta }}}_{A} & = & \sum _{0\leqslant i < j\leqslant 2}\,({\Vert {h}_{i}\Vert }^{2}{\Vert {h}_{j}\Vert }^{2}-{|\langle {h}_{i},{h}_{j}\rangle |}^{2})\end{matrix}$$

with $${p}_{A}=16({{\rm{\Delta }}}_{A}-\tfrac{1}{3})$$, $${\theta }_{A}=\tfrac{1}{3}\,\arccos \,(-\tfrac{{q}_{A}}{2{r}_{A}})$$, $${q}_{A}=\tfrac{64}{3}{{\rm{\Delta }}}_{A}-\tfrac{128}{27}-64det({\rho }_{A}({|\psi \rangle }_{ABC}))$$ and $${r}_{A}=\sqrt{-{(\tfrac{{p}_{A}}{3})}^{3}}$$.

Next we estimate the entanglement between particles *A* and *C*. Let ${{\mathscr{P}}}_{i}:{{\mathbb{C}}}^{9}\to {{\mathbb{C}}}^{9}$ (*i* = 0, 1, 2) be three projections defined, respectively, by

$$\begin{matrix}{{\mathscr{P}}}_{0}{({x}_{1},{x}_{2},{x}_{3},{x}_{4},{x}_{5},{x}_{6},{x}_{7},{x}_{8},{x}_{9})}^{T} & = & {({x}_{1},{x}_{2},{x}_{3},0,0,0,0,0,0)}^{T},\\ {{\mathscr{P}}}_{1}{({x}_{1},{x}_{2},{x}_{3},{x}_{4},{x}_{5},{x}_{6},{x}_{7},{x}_{8},{x}_{9})}^{T} & = & {(0,0,0,{x}_{4},{x}_{5},{x}_{6},0,0,0)}^{T},\\ {{\mathscr{P}}}_{2}{({x}_{1},{x}_{2},{x}_{3},{x}_{4},{x}_{5},{x}_{6},{x}_{7},{x}_{8},{x}_{9})}^{T} & = & {(0,0,0,0,0,0,{x}_{7},{x}_{8},{x}_{9})}^{T},\end{matrix}$$

where ${x}_{j}\in {\mathbb{C}}$ $(j=1,2,\ldots ,9)$. Consider a pure state decomposition of ${\rho }_{AC}({|\psi \rangle }_{ABC})$,

$${\rho }_{AC}({|\psi \rangle }_{ABC})={s}_{0}|{\tau }_{0}\rangle \langle {\tau }_{0}|+{s}_{1}|{\tau }_{1}\rangle \langle {\tau }_{1}|+{s}_{2}|{\tau }_{2}\rangle \langle {\tau }_{2}|$$

with ${s}_{j}={\sum }_{i=0}^{2}\,{\Vert {{\mathscr{P}}}_{j}{h}_{i}\Vert }^{2}$, $|{\tau }_{j}\rangle =\frac{1}{\sqrt{{s}_{j}}}{\sum }_{0\leqslant i,k\leqslant 2}\,{\beta }_{ijk}{|i\rangle }_{A}{|k\rangle }_{C}$ with *j* = 0, 1, 2. Then

$${\rho }_{A}(|{\tau }_{j}\rangle )=\frac{1}{{s}_{j}}\sum _{0\leqslant i,i^{\prime} \leqslant 2}\,\langle {{\mathscr{P}}}_{j}{h}_{i},{{\mathscr{P}}}_{j}{h}_{i}^{\prime} \rangle {|i\rangle }_{A}\langle i^{\prime} |\,(j=0,1,2).$$

Thus, by Lemma 1, we obtain the entanglement degree of |*τ*_*j*_〉 (*j* = 0, 1, 2),

$$\begin{matrix}{ {\mathcal E} }^{M}(|{\tau }_{j}\rangle ) & = & [{(tr\sqrt{{\rho }_{A}(|{\tau }_{j}\rangle )})}^{2}-1]\\  & = & \frac{1}{2}\cdot \frac{{(\sqrt{2+{y}_{{\tau }_{j}}}+\sqrt{2-{y}_{{\tau }_{j}}+2\sqrt{{y}_{{\tau }_{j}}^{2}-4(1-4{{\rm{\Delta }}}_{{\tau }_{j}})}})}^{2}-4}{2},\end{matrix}$$

where

$$\begin{matrix}{y}_{{\tau }_{j}} & = & 2\sqrt{-\frac{{p}_{{\tau }_{j}}}{3}}\,\cos \,{\theta }_{{\tau }_{j}}-\frac{2}{3}\,{\rm{and}}\\ {{\rm{\Delta }}}_{{\tau }_{j}} & = & \frac{1}{{s}_{j}^{2}}[\sum _{0\leqslant i < k\leqslant 2}\,({\Vert {{\mathscr{P}}}_{j}{h}_{i}\Vert }^{2}{\Vert {{\mathscr{P}}}_{j}{h}_{k}\Vert }^{2}-{|\langle {{\mathscr{P}}}_{j}{h}_{i},{{\mathscr{P}}}_{j}{h}_{k}\rangle |}^{2})],\end{matrix}$$

with ${p}_{{\tau }_{j}}=16({{\rm{\Delta }}}_{{\tau }_{j}}-\tfrac{1}{3})$, ${\theta }_{{\tau }_{j}}=\tfrac{1}{3}\,\arccos \,(-\tfrac{{q}_{{\tau }_{j}}}{2{r}_{{\tau }_{j}}})$, ${q}_{{\tau }_{j}}=\tfrac{64}{3}{{\rm{\Delta }}}_{{\tau }_{j}}-\tfrac{128}{27}-64det({\rho }_{A}(|{\tau }_{j}\rangle ))$ and ${r}_{{\tau }_{j}}=\sqrt{-{(\tfrac{{p}_{{\tau }_{j}}}{3})}^{3}}$. Hence, we get a upper bound of the entanglement of ${ {\mathcal E} }_{AC}^{M}$,

$${ {\mathcal E} }_{AC}^{M}={ {\mathcal E} }^{M}[{\rho }_{AC}({|\psi \rangle }_{ABC})]\leqslant \sum _{j=0}^{2}\,{s}_{j}{ {\mathcal E} }^{M}(|{\tau }_{j}\rangle ).$$

Similarly to the above discussion for ${ {\mathcal E} }_{AC}^{M}$, consider a decomposition ${\rho }_{AB}({|\psi \rangle }_{ABC})={\sum }_{l=0}^{2}\,{t}_{l}|{\varsigma }_{l}\rangle \langle {\varsigma }_{l}|$, then ${ {\mathcal E} }_{AB}^{M}={ {\mathcal E} }^{M}[{\rho }_{AB}({|\psi \rangle }_{ABC})]\leqslant {\sum }_{l=0}^{2}\,{t}_{l}{ {\mathcal E} }^{M}(|{\varsigma }_{l}\rangle )$. We might as well assume that

$$\sum _{j=0}^{2}\,{s}_{j}{ {\mathcal E} }^{M}(|{\tau }_{j}\rangle )=\,{\rm{\max }}\{\sum _{j=0}^{2}\,{s}_{j}{ {\mathcal E} }^{M}(|{\tau }_{j}\rangle ),\sum _{l=0}^{2}\,{t}_{l}{ {\mathcal E} }^{M}(|{\varsigma }_{l}\rangle )\}.$$

If we can prove that the following inequality

6 $${ {\mathcal E} }_{A|BC}^{M}\geqslant 2\sum _{j=0}^{2}\,{s}_{j}{ {\mathcal E} }^{M}(|{\tau }_{j}\rangle ),$$

then it will be obtained ${ {\mathcal E} }_{A|BC}^{M}\geqslant { {\mathcal E} }_{AB}^{M}+{ {\mathcal E} }_{AC}^{M}$ and the proof is completed. Next we verify the inequality (8), i.e.,

7 $$\begin{matrix}3[\sqrt{{y}_{A}^{2}-4(1-4{{\rm{\Delta }}}_{A})}+\sqrt{2+{y}_{A}}\sqrt{2-{y}_{A}+2\sqrt{{y}_{A}^{2}-4(1-4{{\rm{\Delta }}}_{A})}}]\\ -\,2\sum _{j=0}^{2}\,{s}_{j}[\sqrt{{y}_{{\tau }_{j}}^{2}-4(1-4{{\rm{\Delta }}}_{{\tau }_{j}})}+\sqrt{2+{y}_{{\tau }_{j}}}\sqrt{2-{y}_{{\tau }_{j}}+2\sqrt{{y}_{{\tau }_{j}}^{2}-4(1-4{{\rm{\Delta }}}_{{\tau }_{j}})}}]\geqslant 0.\end{matrix}$$

Because ${h}_{i}={\sum }_{k=0}^{2}\,{{\mathscr{P}}}_{k}{h}_{i}$ (*i* = 0, 1, 2), we have

$$\begin{matrix}{{\rm{\Delta }}}_{A} & = & \sum _{0\leqslant i < j\leqslant 2}({\Vert \sum _{k=0}^{2}{{\mathscr{P}}}_{k}{h}_{i}\Vert }^{2}{\Vert \sum _{k=0}^{2}{{\mathscr{P}}}_{k}{h}_{j}\Vert }^{2}-{|\langle \sum _{k=0}^{2}{{\mathscr{P}}}_{k}{h}_{i},\sum _{k=0}^{2}{{\mathscr{P}}}_{k}{h}_{j}\rangle |}^{2})\\  & = & \sum _{j=0}^{2}\,{s}_{j}^{2}{{\rm{\Delta }}}_{{\tau }_{j}}+{\lambda }_{{{\rm{\Delta }}}_{A}},\end{matrix}$$

where $${\lambda }_{{{\rm{\Delta }}}_{A}}={\sum }_{\begin{matrix}{\mathfrak{a}},{\mathfrak{b}}\in \mathrm{\{0},1,\mathrm{2\}}\\ {\mathfrak{a}}\ne {\mathfrak{b}}\end{matrix}}\,{\sum }_{0\leqslant i < j\leqslant 2}\,(\langle {{\mathscr{P}}}_{{\mathfrak{a}}}{h}_{i},{{\mathscr{P}}}_{{\mathfrak{a}}}{h}_{i}\rangle \langle {{\mathscr{P}}}_{{\mathfrak{b}}}{h}_{j},{{\mathscr{P}}}_{{\mathfrak{b}}}{h}_{j}\rangle -\langle {{\mathscr{P}}}_{{\mathfrak{a}}}{h}_{i},{{\mathscr{P}}}_{{\mathfrak{a}}}{h}_{j}\rangle \overline{\langle {{\mathscr{P}}}_{{\mathfrak{b}}}{h}_{i},{{\mathscr{P}}}_{{\mathfrak{b}}}{h}_{j}\rangle })$$. Also

$$\begin{matrix}det[{\rho }_{A}({|\psi \rangle }_{ABC})] & = & {\Vert {h}_{0}\Vert }^{2}{\Vert {h}_{1}\Vert }^{2}{\Vert {h}_{2}\Vert }^{2}+\langle {h}_{0},{h}_{1}\rangle \langle {h}_{1},{h}_{2}\rangle \langle {h}_{2},{h}_{0}\rangle \\  &  & +\langle {h}_{1},{h}_{0}\rangle \langle {h}_{2},{h}_{1}\rangle \langle {h}_{0},{h}_{2}\rangle -{\Vert {h}_{1}\Vert }^{2}{|\langle {h}_{0},{h}_{2}\rangle |}^{2}\\  &  & -{\Vert {h}_{2}\Vert }^{2}{|\langle {h}_{0},{h}_{1}\rangle |}^{2}-{\Vert {h}_{0}\Vert }^{2}{|\langle {h}_{1},{h}_{2}\rangle |}^{2}\\  & = & \sum _{j=0}^{2}\,{s}_{j}^{3}det[{\rho }_{A}(|{\tau }_{j}\rangle )]+{\lambda }_{de{t}_{A}},\end{matrix}$$

where

$$\begin{matrix}{\lambda }_{de{t}_{A}} & = & \prod _{i\mathrm{=0}}^{2}\sum _{\begin{matrix}{\mathfrak{a}},{\mathfrak{b}}\in \{0,1,2\}\\ {\mathfrak{a}}\ne {\mathfrak{b}}\end{matrix}}\,\langle {{\mathscr{P}}}_{{\mathfrak{a}}}{h}_{i},{{\mathscr{P}}}_{{\mathfrak{b}}}{h}_{i}\rangle \\  &  & -\,\frac{1}{2}[\sum _{\begin{matrix}i,j,k\in \{0,1,2\}\\ (i-j)(i-k)(j-k)\ne 0\end{matrix}}\,(\sum _{\begin{matrix}{\mathfrak{a}},{\mathfrak{b}}\in \mathrm{\{0},1,\mathrm{2\}}\\ {\mathfrak{a}}\ne {\mathfrak{b}}\end{matrix}}\,\langle {{\mathscr{P}}}_{{\mathfrak{a}}}{h}_{i},{{\mathscr{P}}}_{{\mathfrak{b}}}{h}_{i}\rangle ){|\sum _{\begin{matrix}{\mathfrak{a}},{\mathfrak{b}}\in \mathrm{\{0},1,\mathrm{2\}}\\ {\mathfrak{a}}\ne {\mathfrak{b}}\end{matrix}}\langle {{\mathscr{P}}}_{{\mathfrak{a}}}{h}_{j},{{\mathscr{P}}}_{{\mathfrak{b}}}{h}_{k}\rangle |}^{2}]\\  &  & +\,2Re[(\sum _{\begin{matrix}{\mathfrak{a}},{\mathfrak{b}}\in \mathrm{\{0},1,\mathrm{2\}}\\ {\mathfrak{a}}\ne {\mathfrak{b}}\end{matrix}}\,\langle {{\mathscr{P}}}_{{\mathfrak{a}}}{h}_{0},{{\mathscr{P}}}_{{\mathfrak{b}}}{h}_{1}\rangle )(\sum _{\begin{matrix}{\mathfrak{a}},{\mathfrak{b}}\in \mathrm{\{0},1,\mathrm{2\}}\\ {\mathfrak{a}}\ne {\mathfrak{b}}\end{matrix}}\,\langle {{\mathscr{P}}}_{{\mathfrak{a}}}{h}_{1},{{\mathscr{P}}}_{{\mathfrak{b}}}{h}_{2}\rangle )\\  &  & \times \,(\sum _{\begin{matrix}{\mathfrak{a}},{\mathfrak{b}}\in \mathrm{\{0},1,\mathrm{2\}}\\ {\mathfrak{a}}\ne {\mathfrak{b}}\end{matrix}}\,\langle {{\mathscr{P}}}_{{\mathfrak{a}}}{h}_{2},{{\mathscr{P}}}_{{\mathfrak{b}}}{h}_{0}\rangle )].\end{matrix}$$

Let *λ*_*j*1_, *λ*_*j*2_ and *λ*_*j*3_ be the three non-negative eigenvalues of *s*_*j*_ *ρ*_*A*_(|*τ*_*j*_〉) (*j* = 0, 1, 2), then

8 $${s}_{j}={\lambda }_{j1}+{\lambda }_{j2}+{\lambda }_{j3},$$

9 $${{\rm{\Delta }}}_{{\tau }_{j}}=\frac{{\lambda }_{j1}{\lambda }_{j2}+{\lambda }_{j1}{\lambda }_{j3}+{\lambda }_{j2}{\lambda }_{j3}}{{({\lambda }_{j1}+{\lambda }_{j2}+{\lambda }_{j3})}^{2}},$$

10 $${{\rm{\Delta }}}_{A}=\sum _{j=0}^{2}\,({\lambda }_{j1}{\lambda }_{j2}+{\lambda }_{j1}{\lambda }_{j3}+{\lambda }_{j2}{\lambda }_{j3})+{\lambda }_{{{\rm{\Delta }}}_{A}},$$

11 $$det[{\rho }_{A}(|{\tau }_{j}\rangle )]=\frac{{\lambda }_{j1}{\lambda }_{j2}{\lambda }_{j3}}{{({\lambda }_{j1}+{\lambda }_{j2}+{\lambda }_{j3})}^{3}},$$

12 $$det[{\rho }_{A}({|\psi \rangle }_{ABC})]=\sum _{j=0}^{2}\,({\lambda }_{j1}{\lambda }_{j2}{\lambda }_{j3})+{\lambda }_{de{t}_{A}}.$$

Substituting Eqs (8)–(12) into Eq. (7), we only need to check that the function

13 $$\begin{matrix}f & = & f({\lambda }_{01},{\lambda }_{02},{\lambda }_{03},{\lambda }_{11},{\lambda }_{12},{\lambda }_{13},{\lambda }_{21},{\lambda }_{22},{\lambda }_{{{\rm{\Delta }}}_{A}},{\lambda }_{de{t}_{A}})\\  & = & 3[\sqrt{{y}_{A}^{2}-4(1-4{{\rm{\Delta }}}_{A})}+\sqrt{2+{y}_{A}}\sqrt{2-{y}_{A}+2\sqrt{{y}_{A}^{2}-4(1-4{{\rm{\Delta }}}_{A})}}]\\  &  & -\,2\sum _{j=0}^{2}\,{s}_{j}[\sqrt{{y}_{{\tau }_{j}}^{2}-4(1-4{{\rm{\Delta }}}_{{\tau }_{j}})}\\  &  & +\,\sqrt{2+{y}_{{\tau }_{j}}}\sqrt{2-{y}_{{\tau }_{j}}+2\sqrt{{y}_{{\tau }_{j}}^{2}-4(1-4{{\rm{\Delta }}}_{{\tau }_{j}})}}]\geqslant 0.\end{matrix}$$

Denote ${{\bf{X}}}_{f}=({\lambda }_{01},{\lambda }_{02},{\lambda }_{03},{\lambda }_{11},{\lambda }_{12},{\lambda }_{13},{\lambda }_{21},{\lambda }_{22},{\lambda }_{{{\rm{\Delta }}}_{A}},{\lambda }_{de{t}_{A}})$ and denote the domain of *f* by

$$\begin{matrix}{{\mathscr{D}}}_{f} & = & \{{{\bf{X}}}_{f}|\sum _{i=0}^{2}\sum _{j=1}^{3}\,{\lambda }_{ij}=1\,{\rm{with}}\,{\lambda }_{ij}\geqslant 0,0\leqslant \sum _{j=0}^{2}\,({\lambda }_{j1}{\lambda }_{j2}+{\lambda }_{j1}{\lambda }_{j3}+{\lambda }_{j2}{\lambda }_{j3})\\  &  & +\,{\lambda }_{{{\rm{\Delta }}}_{A}}\leqslant \frac{1}{3},{\lambda }_{{{\rm{\Delta }}}_{A}}\geqslant 0,0\leqslant \sum _{j=0}^{2}\,({\lambda }_{j1}{\lambda }_{j2}{\lambda }_{j3})+{\lambda }_{de{t}_{A}}\leqslant \frac{1}{27},{\lambda }_{de{t}_{A}}\geqslant 0\}.\end{matrix}$$

Then ${{\mathscr{D}}}_{f}$ is a bounded closed set in ${{\mathbb{C}}}^{10}$. More generally, we assume that **X**_*f*_ varies continuously in ${{\mathscr{D}}}_{f}$. Clearly *f* is differential in ${{\mathbb{C}}}^{10}$, and hence *f* has a minimum in ${{\mathscr{D}}}_{f}$. Through a calculation we find that $\frac{\partial f}{\partial {\lambda }_{{{\rm{\Delta }}}_{A}}}\ne 0$ for $0\leqslant {\lambda }_{{{\rm{\Delta }}}_{A}}\leqslant \tfrac{1}{3}$. This implies that there is no stationary point for *f* in the interior of ${{\mathscr{D}}}_{f}$. Hence the minimum of *f* must be achieved on the boundaries of ${{\mathscr{D}}}_{f}$. It can be checked that $f\geqslant 0$ on the boundaries of ${{\mathscr{D}}}_{f}$ (see Supplemental Material). Therefore Eq. (13) holds and the proof is finished.

## Electronic supplementary material

Entanglement monogamy in three qutrit systems
