# Supplementary material for: Entanglement monogamy in three qutrit systems
Source: Sci Rep. 2017 May 16;7:1946. doi: 10.1038/s41598-017-02066-8 (PMC5434069; doi:10.1038/s41598-017-02066-8)
Supplement: Supplementary file 1 — Entanglement monogamy in three qutrit systems [file 41598_2017_2066_MOESM1_ESM.pdf]

Entanglement monogamy in three qutrit systems
Supplemental Material

Qiting Li[†], Jianlian Cui[†] & Shuhao Wang[‡] & Gui-Lu Long[§⊛‡]

[†]Department of Mathematical Sciences, Tsinghua University,

Beijing 100084, P. R. China

[‡]State Key Laboratory of Low-Dimensional Quantum Physics and Department of Physics,

Tsinghua University, Beijing 100084, P. R. China

[§]Tsinghua National Laboratory for Information Science and Technology,

Beijing 100084, P. R. China

[⊛]Collaborative Innovation Center of Quantum Matter,

Beijing 100084, P. R. China

[*] Correspondence to jcui@math.tsinghua.edu.cn

In this Supplemental Material we provide the details of the proofs of the results presented in the main text.

1 Entanglement monogamy in n-qubit systems

Here we prove that for a n-qubit system $\mathcal{H}_1 \otimes \mathcal{H}_2 \otimes \cdots \otimes \mathcal{H}_n$, $\mathcal{E}^M$ satisfies the monogamy inequality, i.e.,

$$\mathcal{E}^M_{1|2\cdots n} \geqslant \mathcal{E}^M_{1|2} + \mathcal{E}^M_{1|3} + \cdots + \mathcal{E}^M_{1|n}. \tag{1}$$

Consider a three partition $\mathscr{A}_3 = A_1|A_2|A_3$ of a n-qubit system $\mathcal{H}_1 \otimes \mathcal{H}_2 \otimes \cdots \otimes \mathcal{H}_n$, where A_1 and A_2 are both single qubit subsystems and A_3 is a $(n-2)$-qubit subsystem. Suppose that we have proved the following inequality for pure states of a $2 \otimes 2 \otimes 2^{n-2}$ system $\mathcal{H}_{A_1} \otimes \mathcal{H}_{A_2} \otimes \mathcal{H}_{A_3}$

$$\mathcal{E}^M_{A_1|A_2A_3} \geqslant \mathcal{E}^M_{A_1A_2} + \mathcal{E}^M_{A_1A_3}. \tag{2}$$

Next we extend the inequality to mixed states $\rho_{A_1|A_2A_3}$ of the system $\mathcal{H}_{A_1} \otimes \mathcal{H}_{A_2} \otimes \mathcal{H}_{A_3}$. Suppose that $\rho_{A_1|A_2A_3} = \sum_i p_i |\psi_i\rangle_{A_1A_2A_3}\langle\psi_i|$ is the minizing decomposition of $\rho_{A_1|A_2A_3}$, applying the inequality (2) to each term, one has

$$\begin{aligned}\mathcal{E}^M(\rho_{A_1|A_2A_3}) = \sum_i p_i \mathcal{E}^M(|\psi_i\rangle_{A_1A_2A_3}) &\geq \sum_i p_i [\mathcal{E}^M(\rho^i_{A_1A_2}) + \mathcal{E}^M(\rho^i_{A_1A_3})] \\ &\geq \mathcal{E}^M(\rho_{A_1A_2}) + \mathcal{E}^M(\rho_{A_1A_3}),\end{aligned}$$

where $\rho^i_{A_1A_2}$ $=\mathrm{tr}_{A_3}(|\psi_i\rangle_{A_1A_2A_3}\langle\psi_i|)$, and the last inequality follows from the inequality (2). For pure states of the n-qubit system $\mathcal{H}_1\otimes\mathcal{H}_2\otimes\cdots\otimes\mathcal{H}_n$, we have

$$\begin{aligned}\mathcal{E}^M_{1|2\cdots n} &\geqslant \mathcal{E}^M_{1|2}+\mathcal{E}^M_{1|3\cdots n}\\ &\geqslant \mathcal{E}^M_{1|2}+\mathcal{E}^M_{1|3}+\mathcal{E}^M_{1|4\cdots n}\\ &\quad\cdots\\ &\geqslant \mathcal{E}^M_{1|2}+\mathcal{E}^M_{1|3}+\cdots+\mathcal{E}^M_{1|(n-1)n}\\ &\geqslant \mathcal{E}^M_{1|2}+\mathcal{E}^M_{1|3}+\cdots+\mathcal{E}^M_{1|(n-1)}+\mathcal{E}^M_{1|n},\end{aligned}$$

where the first inequality follows from the inequality (2) of pure states, the remain inequalities use repeatedly the inequality (2) of mixed states.

Now we prove the inequality (2) for pure states. When $n=3$, it follows from the paragraph perior to the theorem that the inequality holds. We assume $n\geq 4$. Because the reduced density matrices ρ_{A_3} and $\rho_{A_1A_2}$ of the pure state in subsystems A_3 and A_1A_2 have the same rank, and the rank of $\rho_{A_1A_2}$ is not more than 4, by a local unitary invariance, we can regard the 2^{n-2} dimensional qudit A_3 as an effective four dimensional qudit. Thus, to prove the inequality (2), it is sufficient to consider $2\otimes 2\otimes 4$ system $\mathcal{H}_A\otimes\mathcal{H}_B\otimes\mathcal{H}_C$. That is, we need only to prove the following inequality (3) for pure states of a $2\otimes 2\otimes 4$ system $\mathcal{H}_A\otimes\mathcal{H}_B\otimes\mathcal{H}_C$

$$\mathcal{E}^M_{A|BC}\geqslant\mathcal{E}^M_{AB}+\mathcal{E}^M_{AC}. \tag{3}$$

For a general pure state $|\psi\rangle_{ABC}$ in the $2\otimes2\otimes4$ system $\mathcal{H}_A\otimes\mathcal{H}_B\otimes\mathcal{H}_C$, using the Schmidt decomposition, one has

$$|\psi\rangle_{ABC}=\sqrt{p_0}\,|\phi_0\rangle_{AB}\,|0\rangle_C+\sqrt{p_1}\,|\phi_1\rangle_{AB}\,|1\rangle_C+\sqrt{p_2}\,|\phi_2\rangle_{AB}\,|2\rangle_C+\sqrt{p_3}\,|\phi_3\rangle_{AB}\,|3\rangle_C\,,$$

where $\sum_{i=0}^3 p_i=1$ with $0\leqslant p_i\leqslant 1$ $(i=0,1,2,3)$; $\{|\phi_i\rangle_{AB}\mid i=0,1,2,3\}$ is an orthonormal basis of biqubit system $\mathcal{H}_A\otimes\mathcal{H}_B$; $\{|i\rangle_C\mid i=0,1,2,3\}$ is an orthonormal basis of four-dimensional system $\mathcal{H}_C$. According to the Schmidt ranks of $|\phi_i\rangle_{AB}$ $(i=0,1,2,3)$, there are the following five cases.

(1) there is no Schmidt rank-2 state,
(2) there is one Schmidt rank-2 state,
(3) there are two Schmidt rank-2 states,
(4) there are three Schmidt rank-2 states,
(5) there are four Schmidt rank-2 states.

We only need to discuss the last case, i.e., $|\phi_i\rangle_{AB}$, $i=0,1,2,3$, are all Schmidt rank-2 states. Other cases can be similarly proved. With a proper basis $\left\{|\widetilde{0}\rangle_A,|\widetilde{1}\rangle_A\right\}$ and $\left\{|\widetilde{0}\rangle_B,|\widetilde{1}\rangle_B\right\}$ of $\mathcal{H}_A$ and $\mathcal{H}_B$ respectively, $|\psi\rangle_{ABC}$ can be expressed, by Schmidt decomposition, as

$$|\phi_0\rangle_{AB}=a_0|\widetilde{0}\rangle_A|\widetilde{0}\rangle_B+a_3|\widetilde{1}\rangle_A|\widetilde{1}\rangle_B,$$

where a_0 and a_3 are positive numbers with $a_0^2+a_3^2=1$.

Extend $\left\{|\widetilde{0}\rangle_A|\widetilde{0}\rangle_B, |\widetilde{1}\rangle_A|\widetilde{1}\rangle_B\right\}$ to an orthonormal basis of biqubit system $\mathcal{H}_A\otimes\mathcal{H}_B$, denoted by $\left\{|\widetilde{0}\rangle_A|\widetilde{0}\rangle_B, |\widetilde{0}\rangle_A|\widetilde{1}\rangle_B, |\widetilde{1}\rangle_A|\widetilde{0}\rangle_B, |\widetilde{1}\rangle_A|\widetilde{1}\rangle_B\right\}$. Thus, $|\phi_i\rangle_{AB}$ $(i = 1, 2, 3)$ can be expressed as

$$
\begin{aligned}
|\phi_1\rangle_{AB} &= b_0|\widetilde{0}\rangle_A|\widetilde{0}\rangle_B + b_1|\widetilde{0}\rangle_A|\widetilde{1}\rangle_B + b_2|\widetilde{1}\rangle_A|\widetilde{0}\rangle_B + b_3|\widetilde{1}\rangle_A|\widetilde{1}\rangle_B,\\
|\phi_2\rangle_{AB} &= c_0|\widetilde{0}\rangle_A|\widetilde{0}\rangle_B + c_1|\widetilde{0}\rangle_A|\widetilde{1}\rangle_B + c_2|\widetilde{1}\rangle_A|\widetilde{0}\rangle_B + c_3|\widetilde{1}\rangle_A|\widetilde{1}\rangle_B,\\
|\phi_3\rangle_{AB} &= d_0|\widetilde{0}\rangle_A|\widetilde{0}\rangle_B + d_1|\widetilde{0}\rangle_A|\widetilde{1}\rangle_B + d_2|\widetilde{1}\rangle_A|\widetilde{0}\rangle_B + d_3|\widetilde{1}\rangle_A|\widetilde{1}\rangle_B;
\end{aligned}
$$

where b_i, c_i, d_i $(i = 0, 1, 2, 3)$ are complex numbers.

Thus $|\psi\rangle_{ABC}$ can be expressed as

$$
\begin{aligned}
|\psi\rangle_{ABC} \;=\;& \sqrt{p_0}a_0|\widetilde{0}\rangle_A|\widetilde{0}\rangle_B|0\rangle_C + \sqrt{p_1}b_0|\widetilde{0}\rangle_A|\widetilde{0}\rangle_B|1\rangle_C + \sqrt{p_2}c_0|\widetilde{0}\rangle_A|\widetilde{0}\rangle_B|2\rangle_C\\
+\;& \sqrt{p_3}d_0|\widetilde{0}\rangle_A|\widetilde{0}\rangle_B|3\rangle_C + \sqrt{p_1}b_1|\widetilde{0}\rangle_A|\widetilde{1}\rangle_B|1\rangle_C + \sqrt{p_2}c_1|\widetilde{0}\rangle_A|\widetilde{1}\rangle_B|2\rangle_C\\
+\;& \sqrt{p_3}d_1|\widetilde{0}\rangle_A|\widetilde{1}\rangle_B|3\rangle_C + \sqrt{p_1}b_2|\widetilde{1}\rangle_A|\widetilde{0}\rangle_B|1\rangle_C + \sqrt{p_2}c_2|\widetilde{1}\rangle_A|\widetilde{0}\rangle_B|2\rangle_C\\
+\;& \sqrt{p_3}d_2|\widetilde{1}\rangle_A|\widetilde{0}\rangle_B|3\rangle_C + \sqrt{p_0}a_3|\widetilde{1}\rangle_A|\widetilde{1}\rangle_B|0\rangle_C + \sqrt{p_1}b_3|\widetilde{1}\rangle_A|\widetilde{1}\rangle_B|1\rangle_C\\
+\;& \sqrt{p_2}c_3|\widetilde{1}\rangle_A|\widetilde{1}\rangle_B|2\rangle_C + \sqrt{p_3}d_3|\widetilde{1}\rangle_A|\widetilde{1}\rangle_B|3\rangle_C.
\end{aligned}
$$

One can calculate that

$$\rho_A(|\psi\rangle_{ABC}) = \xi_1|\widetilde{0}\rangle_A\langle\widetilde{0}| + \xi_2|\widetilde{0}\rangle_A\langle\widetilde{1}| + \xi_3|\widetilde{1}\rangle_A\langle\widetilde{0}| + \xi_4|\widetilde{1}\rangle_A\langle\widetilde{1}|,$$

where

$$
\begin{aligned}
\xi_1 &= p_0a_0^2 + p_1|b_0|^2 + p_1|b_1|^2 + p_2|c_0|^2 + p_2|c_1|^2 + p_3|d_0|^2 + p_3|d_1|^2,\\
\xi_2 &= p_1b_0\overline{b_2} + p_1b_1\overline{b_3} + p_2c_0\overline{c_2} + p_2c_1\overline{c_3} + p_3d_0\overline{d_2} + p_3d_1\overline{d_3},\\
\xi_3 &= \overline{\xi_2},\\
\xi_4 &= p_0a_3^2 + p_1|b_2|^2 + p_1|b_3|^2 + p_2|c_2|^2 + p_2|c_3|^2 + p_3|d_2|^2 + p_3|d_3|^2,
\end{aligned}
$$

with $\overline{\omega_\nu}$ is complex conjugate of ω_ν. It follows that the entanglement between the particle A and the particles BC

$$
\begin{aligned}
\mathcal{E}^M_{A|BC} \;=\;& \frac{8}{3}\left[\left(tr\sqrt{\rho_A(|\psi\rangle_{ABC})}\right)^2 - 1\right]\\
=\;& \frac{16}{3}\sqrt{\xi_1\xi_4 - \xi_2\xi_3}\\
=\;& \frac{16}{3}\big(p_1p_2|b_0c_2 - c_0b_2|^2 + p_1p_2|b_0c_3 - c_1b_2|^2 + p_1^2|b_0b_3 - b_1b_2|^2\\
& +p_1p_3|b_0d_2 - b_2d_0|^2 + p_1p_3|b_0d_3 - b_2d_1|^2 + p_1p_2|c_2d_1 - c_0d_3|^2\\
& +p_1p_3|b_1d_2 - b_3d_0|^2 + p_1p_3|b_1d_3 - b_3d_1|^2 + p_2p_3|c_0d_3 - c_2d_1|^2\\
& +p_2p_3|c_0d_2 - c_2d_0|^2 + p_1p_2|b_1c_3 - c_1b_3|^2 + p_2^2|c_1c_2 - c_0c_3|^2\\
& +p_2p_3|c_1d_3 - c_3d_1|^2 + p_2p_3|c_1d_2 - c_3d_0|^2 + p_3^2|d_0d_3 - d_1d_2|^2\\
& +p_1p_2|c_0b_3 - c_2b_1|^2 + p_0^2a_0^2a_3^2 + p_0p_1a_0^2|b_2|^2 + p_0p_1a_0^2|b_3|^2 + p_0p_2a_0^2|c_2|^2\\
& +p_0p_2a_0^2|c_3|^2 + p_0p_3a_0^2|d_2|^2 + p_0p_3a_0^2|d_3|^2 + p_0p_1a_3^2|b_0|^2 + p_0p_1a_3^2|b_1|^2\\
& +p_0p_2a_3^2|c_0|^2 + p_0p_2a_3^2|c_1|^2 + p_0p_3a_3^2|d_0|^2 + p_0p_3a_3^2|d_1|^2\;\big)^{\frac{1}{2}}.
\end{aligned}
$$

Next, we calculate the entanglement between particles A and C. For the sake of simplicity, write $|\widetilde{i}\rangle_A|j\rangle_C = |e_{ij}\rangle$ where $i = 0, 1$ and $j = 0, 1, 2, 3$. Consider a pure state decomposition of $\rho_{AC}(|\psi\rangle_{ABC})$,

$$\rho_{AC}(|\psi\rangle_{ABC}) = r_1|\varphi_1\rangle\langle\varphi_1| + r_2|\varphi_2\rangle\langle\varphi_2|,$$

where

$$\begin{aligned}
r_1 &= p_0a_0^2 + p_1|b_0|^2 + p_1|b_2|^2 + p_2|c_0|^2 + p_2|c_2|^2 + p_3|d_0|^2 + p_3|d_2|^2,\\
r_2 &= p_0a_3^2 + p_1|b_1|^2 + p_1|b_3|^2 + p_2|c_1|^2 + p_2|c_3|^2 + p_3|d_1|^2 + p_3|d_3|^2,\\
|\varphi_1\rangle &= \frac{1}{\sqrt{p_0a_0^2 + p_1|b_0|^2 + p_1|b_2|^2 + p_2|c_0|^2 + p_2|c_2|^2 + p_3|d_0|^2 + p_3|d_2|^2}} \left(\sqrt{p_0}a_0|e_{00}\rangle\right.\\
&\quad \left. + \sqrt{p_1}b_0|e_{01}\rangle + \sqrt{p_2}c_0|e_{02}\rangle + \sqrt{p_3}d_0|e_{03}\rangle + \sqrt{p_1}b_2|e_{11}\rangle + \sqrt{p_2}c_2|e_{12}\rangle + \sqrt{p_3}d_2|e_{13}\rangle\right),\\
|\varphi_2\rangle &= \frac{1}{\sqrt{p_0a_3^2 + p_1|b_1|^2 + p_1|b_3|^2 + p_2|c_1|^2 + p_2|c_3|^2 + p_3|d_1|^2 + p_3|d_3|^2}} \left(\sqrt{p_1}b_1|e_{01}\rangle\right.\\
&\quad \left. + \sqrt{p_2}c_1|e_{02}\rangle + \sqrt{p_3}d_1|e_{03}\rangle + \sqrt{p_0}a_3|e_{10}\rangle + \sqrt{p_1}b_3|e_{11}\rangle + \sqrt{p_2}c_3|e_{12}\rangle + \sqrt{p_3}d_3|e_{13}\rangle\right).
\end{aligned}$$

Then

$$\rho_A(|\varphi_1\rangle) = y_{1,1}|\widetilde{0}\rangle_A\langle\widetilde{0}| + y_{1,2}|\widetilde{0}\rangle_A\langle\widetilde{1}| + y_{1,3}|\widetilde{1}\rangle_A\langle\widetilde{0}| + y_{1,4}|\widetilde{1}\rangle_A\langle\widetilde{1}|,$$

here

$$\begin{aligned}
y_{1,1} &= \frac{p_0a_0^2 + p_1|b_0|^2 + p_2|c_0|^2 + p_3|d_0|^2}{p_0a_0^2 + p_1|b_0|^2 + p_1|b_2|^2 + p_2|c_0|^2 + p_2|c_2|^2 + p_3|d_0|^2 + p_3|d_2|^2},\\
y_{1,2} &= \frac{p_1b_0\overline{b_2} + p_2c_0\overline{c_2} + p_3d_0\overline{d_2}}{p_0a_0^2 + p_1|b_0|^2 + p_1|b_2|^2 + p_2|c_0|^2 + p_2|c_2|^2 + p_3|d_0|^2 + p_3|d_2|^2},\\
y_{1,3} &= \overline{y_{1,2}},\\
y_{1,4} &= \frac{p_1|b_2|^2 + p_2|c_2|^2 + p_3|d_2|^2}{p_0a_0^2 + p_1|b_0|^2 + p_1|b_2|^2 + p_2|c_0|^2 + p_2|c_2|^2 + p_3|d_0|^2 + p_3|d_2|^2}.
\end{aligned}$$

Hence

$$\mathcal{E}^M(|\varphi_1\rangle) = \left[\left(tr\sqrt{\rho_A(|\varphi_1\rangle)}\right)^2 - 1\right] = 2\sqrt{y_{1,1}y_{1,4} - y_{1,2}y_{1,3}}. \tag{4}$$

Similarly,

$$\rho_A(|\varphi_2\rangle) = y_{2,1}|\widetilde{0}\rangle_A\langle\widetilde{0}| + y_{2,2}|\widetilde{0}\rangle_A\langle\widetilde{1}| + y_{2,3}|\widetilde{1}\rangle_A\langle\widetilde{0}| + y_{2,4}|\widetilde{1}\rangle_A\langle\widetilde{1}|,$$

where

$$\begin{aligned}
y_{2,1} &= \frac{p_1|b_1|^2 + p_2|c_1|^2 + p_3|d_1|^2}{p_0a_3^2 + p_1|b_1|^2 + p_1|b_3|^2 + p_2|c_1|^2 + p_2|c_3|^2 + p_3|d_1|^2 + p_3|d_3|^2},\\
y_{2,2} &= \frac{p_1b_1\overline{b_3} + p_2c_1\overline{c_3} + p_3d_1\overline{d_3}}{p_0a_3^2 + p_1|b_1|^2 + p_1|b_3|^2 + p_2|c_1|^2 + p_2|c_3|^2 + p_3|d_1|^2 + p_3|d_3|^2},\\
y_{2,3} &= \overline{y_{2,2}},\\
y_{2,4} &= \frac{p_0|a_3|^2 + p_1|b_3|^2 + p_2|c_3|^2 + p_3|d_3|^2}{p_0a_3^2 + p_1|b_1|^2 + p_1|b_3|^2 + p_2|c_1|^2 + p_2|c_3|^2 + p_3|d_1|^2 + p_3|d_3|^2}.
\end{aligned}$$

Therefore,

$$\mathcal{E}^M(|\varphi_2\rangle) = \left[\left(tr\sqrt{\rho_A(|\varphi_2\rangle)}\right)^2 - 1\right] = 2\sqrt{y_{2,1}y_{2,4} - y_{2,2}y_{2,3}}. \tag{5}$$

Thus, by Eq. (4) and Eq. (5), we have

$$\begin{aligned}
\mathcal{E}^M_{AC} &= \mathcal{E}^M\left[\rho_{AC}(|\psi\rangle_{ABC})\right] \\
&\leqslant r_1\mathcal{E}^M(|\varphi_1\rangle) + r_2\mathcal{E}^M(|\varphi_2\rangle) \\
&= 2\left(r_1\sqrt{y_{1,1}y_{1,4} - y_{1,2}y_{1,3}} + r_2\sqrt{y_{2,1}y_{2,4} - y_{2,2}y_{2,3}}\right) \\
&= 2\left[\left(p_1p_2|b_0c_2 - c_0b_2|^2 + p_1p_3|b_0d_2 - b_2d_0|^2 + p_2p_3|c_0d_2 - c_2d_0|^2 + p_0p_1a_0^2|b_2|^2\right.\right. \\
&\quad \left. + p_0p_2a_0^2|c_2|^2 + p_0p_3a_0^2|d_2|^2\right)^{\frac{1}{2}} + \left(p_0p_1a_3^2|b_1|^2 + p_0p_2a_3^2|c_1|^2 + p_0p_3a_3^2|d_1|^2\right. \\
&\quad \left.\left. + p_1p_2|b_1c_3 - c_1b_3|^2 + p_2p_3|c_1d_3 - c_3d_1|^2 + p_1p_3|b_1d_3 - b_3d_1|^2\right)^{\frac{1}{2}}\ \right].
\end{aligned}$$

For the sake of simplicity, let

$$\begin{aligned}
\varrho_1 &= \left(p_1p_2|b_0c_2 - c_0b_2|^2 + p_1p_3|b_0d_2 - b_2d_0|^2 + p_2p_3|c_0d_2 - c_2d_0|^2 + p_0p_1a_0^2|b_2|^2 + p_0p_2a_0^2|c_2|^2\right. \\
&\quad \left. + p_0p_3a_0^2|d_2|^2\right)^{\frac{1}{2}}, \\
\varrho_2 &= \left(p_0p_1a_3^2|b_1|^2 + p_0p_2a_3^2|c_1|^2 + p_0p_3a_3^2|d_1|^2 + p_1p_2|b_1c_3 - c_1b_3|^2 + p_2p_3|c_1d_3 - c_3d_1|^2\right. \\
&\quad \left. + p_1p_3|b_1d_3 - b_3d_1|^2\right)^{\frac{1}{2}}.
\end{aligned}$$

Then $\mathcal{E}^M_{AC} \leqslant 2(\varrho_1 + \varrho_2)$.

It remains to calculate $\mathcal{E}^M_{AB}$. It is clear that

$$\rho_{AB}(|\psi\rangle_{ABC}) = p_0|\phi_0\rangle_{AB}\langle\phi_0| + p_1|\phi_1\rangle_{AB}\langle\phi_1| + p_2|\phi_2\rangle_{AB}\langle\phi_2| + p_3|\phi_3\rangle_{AB}\langle\phi_3|.$$

Then $\rho_A(|\phi_0\rangle_{AB}) = a_0^2|\widetilde{0}\rangle_A\langle\widetilde{0}| + a_3^2|\widetilde{1}\rangle_A\langle\widetilde{1}|$, and

$$\mathcal{E}^M(|\phi_0\rangle_{AB}) = 2a_0a_3. \tag{6}$$

Similarly, for $i = 1, 2, 3$, we obtain that

$$\rho_A(|\phi_i\rangle_{AB}) \;=\; z_{i,1}|\widetilde{0}\rangle_A\langle\widetilde{0}| + z_{i,2}|\widetilde{0}\rangle_A\langle\widetilde{1}| + z_{i,3}|\widetilde{1}\rangle_A\langle\widetilde{0}| + z_{i,4}|\widetilde{1}\rangle_A\langle\widetilde{1}|,$$

where $z_{1,1} = |b_0|^2 + |b_1|^2$, $z_{1,2} = b_0\overline{b_2} + b_1\overline{b_3}$, $z_{1,3} = \overline{z_{1,2}}$, $z_{1,4} = |b_2|^2 + |b_3|^2$, $z_{2,1} = |c_0|^2 + |c_1|^2$, $z_{2,2} = c_0\overline{c_2} + c_1\overline{c_3}$, $z_{2,3} = \overline{z_{2,2}}$, $z_{2,4} = |c_2|^2 + |c_3|^2$, $z_{3,1} = |d_0|^2 + |d_1|^2$, $z_{3,2} = d_0\overline{d_2} + d_1\overline{d_3}$, $z_{3,3} = \overline{z_{3,2}}$, $z_{3,4} = |d_2|^2 + |d_3|^2$. It can be calculated that

$$\begin{aligned}
\mathcal{E}^M(|\phi_1\rangle_{AB}) &= 2\sqrt{z_{1,1}z_{1,4} - z_{1,2}z_{1,3}}, \qquad (7) \\
\mathcal{E}^M(|\phi_2\rangle_{AB}) &= 2\sqrt{z_{2,1}z_{2,4} - z_{2,2}z_{2,3}}, \qquad (8) \\
\mathcal{E}^M(|\phi_3\rangle_{AB}) &= 2\sqrt{z_{3,1}z_{3,4} - z_{3,2}z_{3,3}}. \qquad (9)
\end{aligned}$$

By Eq. (6), Eq. (7), Eq. (8) and Eq. (9), one has

$$\begin{aligned}
\mathcal{E}_{AB}^{M} &= \mathcal{E}^{M}\left[\rho_{AB}(|\psi\rangle_{ABC})\right] \\
&\leqslant p_0\mathcal{E}^{M}(|\phi_1\rangle_{AB}) + p_1\mathcal{E}^{M}(|\phi_2\rangle_{AB}) + p_2\mathcal{E}^{M}(|\phi_3\rangle_{AB}) + p_3\mathcal{E}^{M}(|\phi_4\rangle_{AB}) \\
&= 2\left(p_0a_0a_3 + p_1\sqrt{z_{1,1}z_{1,4} - z_{1,2}z_{1,3}} + p_2\sqrt{z_{2,1}z_{2,4} - z_{2,2}z_{2,3}} + p_3\sqrt{z_{3,1}z_{3,4} - z_{3,2}z_{3,3}}\right) \\
&= 2\left(p_0a_0a_3 + p_1|b_0b_3 - b_1b_2| + p_2|c_0c_3 - c_1c_2| + p_3|d_0d_3 - d_1d_2|\right).
\end{aligned}$$

For simplicity, let $\varrho_3 = p_0a_0a_3$, $\varrho_4 = p_1|b_0b_3 - b_1b_2|$, $\varrho_5 = p_2|c_0c_3 - c_1c_2|$ and $\varrho_6 = p_3|d_0d_3 - d_1d_2|$. Then $\mathcal{E}_{AB}^{M} \leqslant 2\left(\varrho_3 + \varrho_4 + \varrho_5 + \varrho_6\right)$. In addition, let

$$\begin{aligned}
\varrho_7 &= p_1p_2|b_0c_3 - c_1b_2|^2 + p_1p_3|b_0d_3 - b_2d_1|^2 + p_1p_2|c_2d_1 - c_0d_3|^2 + p_1p_3|b_1d_2 - b_3d_0|^2 \\
&\quad + p_2p_3|c_0d_3 - c_2d_1|^2 + p_2p_3|c_1d_2 - c_3d_0|^2 + p_1p_2|c_0b_3 - c_2b_1|^2 + p_0p_1a_0^2|b_3|^2 \\
&\quad + p_0p_2a_0^2|c_3|^2 + p_0p_3a_0^2|d_3|^2 + p_0p_1a_3^2|b_0|^2 + p_0p_2a_3^2|c_0|^2 + p_0p_3a_3^2|d_0|^2.
\end{aligned}$$

Hence,

$$\mathcal{E}_{A|BC}^{M} = \frac{16}{3}\left(\varrho_1^2 + \varrho_2^2 + \varrho_3^2 + \varrho_4^2 + \varrho_5^2 + \varrho_6^2 + \varrho_7\right)^{\frac{1}{2}}.$$

Thus, if we can prove that the following inequality

$$\frac{8}{3}\left(\varrho_1^2 + \varrho_2^2 + \varrho_3^2 + \varrho_4^2 + \varrho_5^2 + \varrho_6^2 + \varrho_7\right)^{\frac{1}{2}} \geqslant \varrho_1 + \varrho_2 + \varrho_3 + \varrho_4 + \varrho_5 + \varrho_6,$$

then it follows that $\mathcal{E}_{A|BC}^{M} \geqslant \mathcal{E}_{AB}^{M} + \mathcal{E}_{AC}^{M}$.

Note that $0 \leqslant \varrho_1 + \varrho_2 \leqslant \frac{1}{2}$, $0 \leqslant \sum_{i=3}^{6}\varrho_i \leqslant \frac{1}{2}$ and $0 \leqslant \sum_{i=1}^{6}\varrho_i^2 + \varrho_7 \leqslant \frac{1}{4}$, thus we can regard ϱ_i $(1 \leqslant i \leqslant 7)$ as independent variables. Let

$$\begin{aligned}
h &= h\left(\varrho_1, \varrho_2, \varrho_3, \varrho_4, \varrho_5, \varrho_6, \varrho_7\right) \\
&= \frac{8}{3}\left(\varrho_1^2 + \varrho_2^2 + \varrho_3^2 + \varrho_4^2 + \varrho_5^2 + \varrho_6^2 + \varrho_7\right)^{\frac{1}{2}} - \varrho_1 - \varrho_2 - \varrho_3 - \varrho_4 - \varrho_5 - \varrho_6.
\end{aligned}$$

Denote $\mathbf{X}_h = (\varrho_1, \varrho_2, \varrho_3, \varrho_4, \varrho_5, \varrho_6, \varrho_7)$ and denote the domain of h by

$$\mathcal{D}_h = \left\{\mathbf{X}_h|\varrho_i \geqslant 0(1 \leqslant i \leqslant 7), 0 \leqslant \varrho_1 + \varrho_2 \leqslant \frac{1}{2}, 0 \leqslant \sum_{i=3}^{6}\varrho_i \leqslant \frac{1}{2} \text{ and } 0 \leqslant \sum_{i=1}^{6}\varrho_i^2 + \varrho_7 \leqslant \frac{1}{4}\right\}.$$

Then $\mathcal{D}_h$ is a bounded closed set in $\mathbb{C}^7$. More generally, we assume that $\mathbf{X}_h$ varies continuously in $\mathcal{D}_h$. Clearly h is differential in $\mathbb{C}^7$, and hence h has a minimum in $\mathcal{D}_h$. Because $\frac{\partial h}{\partial \varrho_7} \neq 0$, there is no stationary point for h in the interior of $\mathcal{D}_h$. Consequently, the minimum of h must be achieved on the boundaries of $\mathcal{D}_h$. Next we check that $h \geqslant 0$ on the boundaries

of $\mathcal{D}_h$. We divide the proof into the following several cases based on the boundaries:

$$\begin{aligned}
&\textbf{Case 1}\ \ \partial_{\sum_{i=1}^{6}\varrho_i^2+\varrho_7=\frac{1}{4}}\mathcal{D}_h=\left\{\mathbf{X}_h\in\mathcal{D}_h|\sum_{i=1}^{6}\varrho_i^2+\varrho_7=\frac{1}{4}\right\},\\
&\textbf{Case 2}\ \ \partial_{\varrho_7=0}\mathcal{D}_h=\left\{\mathbf{X}_h\in\mathcal{D}_h|\varrho_7=0\right\},\\
&\textbf{Case 3}\ \ \partial_{\varrho_i=0}\mathcal{D}_h=\left\{\mathbf{X}_h\in\mathcal{D}_h|\varrho_i=0\right\},1\leqslant i\leqslant 6,\\
&\textbf{Case 4}\ \ \partial_{\varrho_1+\varrho_2=\frac{1}{2}}\mathcal{D}_h=\left\{\mathbf{X}_h\in\mathcal{D}_h|\varrho_1+\varrho_2=\frac{1}{2}\right\},\\
&\textbf{Case 5}\ \ \partial_{\sum_{i=3}^{6}\varrho_i=\frac{1}{2}}\mathcal{D}_h=\left\{\mathbf{X}_h\in\mathcal{D}_h|\sum_{i=3}^{6}\varrho_i=\frac{1}{2}\right\}.
\end{aligned}$$

1.1 Case 1

In this case, we have

$$\begin{aligned}
h(\varrho_1,\varrho_2,\varrho_3,\varrho_4,\varrho_5,\varrho_6,\varrho_7) &= \frac{8}{3}\sqrt{\frac{1}{4}}-\varrho_1-\varrho_2-\varrho_3-\varrho_4-\varrho_5-\varrho_6\\
&\geqslant \frac{4}{3}-\frac{1}{2}-\frac{1}{2}=\frac{1}{3}>0.
\end{aligned}$$

1.2 Case 2

In this case, let

$$\begin{aligned}
h_2 &= h(\varrho_1,\varrho_2,\varrho_3,\varrho_4,\varrho_5,\varrho_6,0)\\
&= \frac{8}{3}\left(\varrho_1^2+\varrho_2^2+\varrho_3^2+\varrho_4^2+\varrho_5^2+\varrho_6^2\right)^{\frac{1}{2}}-\varrho_1-\varrho_2-\varrho_3-\varrho_4-\varrho_5-\varrho_6.
\end{aligned}$$

By a direct computation, it can be obtained that there is no stationary point for h_2 in $\partial_{\varrho_7=0}\mathcal{D}_h$. So the minimum of h_2 must be achieved on the boundary of $\partial_{\varrho_7=0}\mathcal{D}_h$. We consider the following subcases:

$$\begin{aligned}
&\textbf{Subcase 2(a)}\ \ \partial_{\varrho_i=0}\left(\partial_{\varrho_7=0}\mathcal{D}_h\right)=\left\{\mathbf{X}_h\in\mathcal{D}_h|\varrho_7=0\text{ and }\varrho_i=0\right\},1\leqslant i\leqslant 6,\\
&\textbf{Subcase 2(b)}\ \ \partial_{\varrho_1+\varrho_2=\frac{1}{2}}\left(\partial_{\varrho_7=0}\mathcal{D}_h\right)=\left\{\mathbf{X}_h\in\mathcal{D}_h|\varrho_7=0\text{ and }\varrho_1+\varrho_2=\frac{1}{2}\right\},\\
&\textbf{Subcase 2(c)}\ \ \partial_{\sum_{i=3}^{6}\varrho_i=\frac{1}{2}}\left(\partial_{\varrho_7=0}\mathcal{D}_h\right)=\left\{\mathbf{X}_h\in\mathcal{D}_h|\varrho_7=0\text{ and }\sum_{i=3}^{6}\varrho_i=\frac{1}{2}\right\}.
\end{aligned}$$

1.2.1 Subcase 2(a)

Through the above analysis similar to Case 2, the problem is reduced to the case that only one variable is nonzero, for example, ϱ_1, then, clearly $h(\varrho_1,0,0,0,0,0,0)=\frac{5}{3}\varrho_1\geqslant 0$.

1.2.2 Subcase 2(b)

Consider

$$\begin{aligned}
h_{2(b)} &= h(\varrho_2,\varrho_3,\varrho_4,\varrho_5,\varrho_6,0)\\
&= \frac{8}{3}\left[\left(\frac{1}{2}-\varrho_2\right)^2+\varrho_2^2+\varrho_3^2+\varrho_4^2+\varrho_5^2+\varrho_6^2\right]^{\frac{1}{2}}-\frac{1}{2}-\varrho_3-\varrho_4-\varrho_5-\varrho_6.
\end{aligned}$$

It can be computed that there is no stationary point for $h_{2(b)}$ in $\partial_{\varrho_1+\varrho_2=\frac{1}{2}}\left(\partial_{\varrho_7=0}\mathcal{D}_h\right)$. Hence the minimum of $h_{2(b)}$ is achieved on the boundary of $\partial_{\varrho_1+\varrho_2=\frac{1}{2}}\left(\partial_{\varrho_7=0}\mathcal{D}_h\right)$. According to Subcase 2(a), we only need to evaluate $h_{2(b)}$ on $\partial_{\sum_{i=3}^{6}\varrho_i=\frac{1}{2}}\left(\partial_{\varrho_1+\varrho_2=\frac{1}{2}}\left(\partial_{\varrho_7=0}\mathcal{D}_h\right)\right)$. Because

$$\begin{aligned} & \frac{8}{3}\left(\varrho_1^2+\varrho_2^2+\varrho_3^2+\varrho_4^2+\varrho_5^2+\varrho_6^2\right)^{\frac{1}{2}}-\left(\varrho_1+\varrho_2+\varrho_3+\varrho_4+\varrho_5+\varrho_6\right) \\ \geqslant & \frac{8}{3}\left(\varrho_1^2+\varrho_2^2+\varrho_3^2+\varrho_4^2+\varrho_5^2+\varrho_6^2\right)^{\frac{1}{2}}-1 \\ \geqslant & \frac{8}{3}\left[\frac{1}{2}\left(\varrho_1+\varrho_2\right)^2+\frac{1}{4}\left(\varrho_3+\varrho_4+\varrho_5+\varrho_6\right)^2\right]^{\frac{1}{2}}-1 \\ = & \frac{2\sqrt{3}}{3}-1>0, \end{aligned}$$

we have $h_{2(b)}>0$.

1.2.3 Subcase 2(c)

Let $\varrho_{12}=\varrho_1+\varrho_2$, then $0\leqslant\varrho_{12}\leqslant\frac{1}{2}$. It follows that

$$\begin{aligned} h_{2(c)} &= h\left(\varrho_1,\varrho_2,\varrho_4,\varrho_5,\varrho_6,0\right) \\ &= \frac{8}{3}\left(\varrho_1^2+\varrho_2^2+\varrho_3^2+\varrho_4^2+\varrho_5^2+\varrho_6^2\right)^{\frac{1}{2}}-\left(\varrho_1+\varrho_2\right)-\frac{1}{2} \\ &\geqslant \frac{8}{3}\left[\frac{1}{2}\left(\varrho_1+\varrho_2\right)^2+\frac{1}{4}\left(\varrho_3+\varrho_4+\varrho_5+\varrho_6\right)^2\right]^{\frac{1}{2}}-\left(\varrho_1+\varrho_2\right)-\frac{1}{2} \\ &= \frac{8}{3}\left(\frac{1}{2}\varrho_{12}^2+\frac{1}{16}\right)^{\frac{1}{2}}-\varrho_{12}-\frac{1}{2} \\ &> 0. \end{aligned}$$

1.3 Case 3

Without loss of generalization, we assume that $\varrho_1=0$. Let

$$\begin{aligned} h_3 &= h\left(0,\varrho_2,\varrho_3,\varrho_4,\varrho_5,\varrho_6,\varrho_7\right) \\ &= \frac{8}{3}\left(\varrho_2^2+\varrho_3^2+\varrho_4^2+\varrho_5^2+\varrho_6^2+\varrho_7\right)^{\frac{1}{2}}-\varrho_2-\varrho_3-\varrho_4-\varrho_5-\varrho_6. \end{aligned}$$

Because $\frac{\partial h_3}{\partial\varrho_7}\neq 0$, there is no stationary point for h_3 in the interior of $\partial_{\varrho_1=0}\mathcal{D}_h$. Thus the minimum of h_3 must be achieved on the boundaries of $\partial_{\varrho_1=0}\mathcal{D}_h$. Repeating the process above, the problem under consideration is reduced to proving $h\left(0,0,0,0,0,0,\varrho_7\right)=\frac{8}{3}\sqrt{\varrho_7}\geqslant 0$, it is clear.

1.4 Case 4

In this case, a similar discussion just as Subcase 2(c) implies that

$$\begin{aligned} h_4 &= h\left(\varrho_1,\varrho_2,\varrho_3,\varrho_4,\varrho_5,\varrho_6,\varrho_7\right) \\ &= \frac{8}{3}\left[\varrho_1^2+\varrho_2^2+\varrho_3^2+\varrho_4^2+\varrho_5^2+\varrho_6^2+\varrho_7\right]^{\frac{1}{2}}-\frac{1}{2}-\varrho_3-\varrho_4-\varrho_5-\varrho_6 \\ &\geqslant \frac{8}{3}\left(\frac{1}{8}+\frac{1}{4}(\varrho_3+\varrho_4+\varrho_5+\varrho_6)^2\right)^{\frac{1}{2}}-\frac{1}{2}-(\varrho_3+\varrho_4+\varrho_5+\varrho_6), \\ &> 0. \end{aligned}$$

1.5 Case 5

In this case, it follows that

$$\begin{aligned} h_5 &= h\left(\varrho_1, \varrho_2, \varrho_3, \varrho_4, \varrho_5, \varrho_6, \varrho_7\right) \\ &\geqslant \frac{8}{3}\left[\frac{1}{2}(\varrho_1+\varrho_2)^2+\frac{1}{16}\right]^{\frac{1}{2}} - (\varrho_1+\varrho_2) - \frac{1}{2} \\ &> 0. \end{aligned}$$

The proof is completed.

2 The computable formula for $\mathcal{E}^M$

Here we prove the Lemma 1. By Cauchy-Schwarz inequality we know that

$$\Delta = \sum_{1\leqslant i<j\leqslant 3} \|\alpha_i\|^2\|\alpha_j\|^2 - \sum_{1\leqslant i<j\leqslant 3} |\langle\alpha_i, \alpha_j\rangle|^2 \geqslant 0.$$

Furthermore, it can be seen that $\Delta \leqslant \sum_{1\leqslant i<j\leqslant 3} \|\alpha_i\|^2\|\alpha_j\|^2 \leqslant \frac{1}{3}$. Let λ_1, λ_2 and λ_3 be the eigenvalues of ρ, then we have $0 \leqslant det(\rho) = \lambda_1\lambda_2\lambda_3 \leqslant \frac{1}{27}$. Set $\mathcal{X} = tr\sqrt{\rho}$, then

$$\begin{aligned} \left(\mathcal{X}^2-1\right)^2 &= \left[(tr\sqrt{\rho})^2-1\right]^2 \\ &= 4\left[\sqrt{\lambda_1\lambda_2}+\sqrt{\lambda_1\lambda_3}+\sqrt{\lambda_2\lambda_3}\right]^2 \\ &= 4\left[\Delta+2\sqrt{det(\rho)}\left(\sqrt{\lambda_1}+\sqrt{\lambda_2}+\sqrt{\lambda_3}\right)\right] \\ &= 4\left[\Delta+2\sqrt{det(\rho)}\mathcal{X}\right]. \end{aligned}$$

Therefore, to get the analytical expression of $tr\sqrt{\rho}$, we need to solve the following quartic equation

$$\mathcal{X}^4 - 2\mathcal{X}^2 - 8\sqrt{det(\rho)}\mathcal{X} + 1 - 4\Delta = 0, \tag{10}$$

whose four roots equal to that of the following two quadratic equations

$$\mathcal{X}^2 + \sqrt{2+y}\mathcal{X} + \frac{1}{2}\left[y+\sqrt{y^2-4(1-4\Delta)}\right] = 0 \tag{11}$$

and

$$\mathcal{X}^2 - \sqrt{2+y}\mathcal{X} + \frac{1}{2}\left[y-\sqrt{y^2-4(1-4\Delta)}\right] = 0, \tag{12}$$

where y is an arbitrary real root of the following cubic equation

$$\mathcal{Y}^3 + 2\mathcal{Y}^2 - 4\left(1-4\Delta\right)\mathcal{Y} - 8\left(1-4\Delta\right) - 64det(\rho) = 0. \tag{13}$$

To solve Eq. (13), let $\mathcal{Y} = \mathcal{Z} - \frac{2}{3}$, then we have

$$\mathcal{Z}^3 + 16\left(\Delta-\frac{1}{3}\right)\mathcal{Z} + \frac{64}{3}\Delta - \frac{128}{27} - 64det(\rho) = 0. \tag{14}$$

Because $-\frac{2}{5} < \left(\frac{q}{2}\right)^2 + \left(\frac{p}{3}\right)^3 \leqslant 0$, the roots of Eq. (14) are

$$\begin{aligned} z_1 &= 2\sqrt{-\frac{p}{3}}\cos\theta, \\ z_2 &= 2\sqrt{-\frac{p}{3}}\cos(\theta + \frac{2\pi}{3}), \\ z_3 &= 2\sqrt{-\frac{p}{3}}\cos(\theta + \frac{4\pi}{3}), \end{aligned}$$

where $p = 16\left(\Delta - \frac{1}{3}\right)$, $\theta = \frac{1}{3}\arccos\left(-\frac{q}{2r}\right)$, $q = \frac{64}{3}\Delta - \frac{128}{27} - 64det(\rho)$ and $r = \sqrt{-\left(\frac{p}{3}\right)^3}$.

We might as well take $y = z_1 - \frac{2}{3}$. Now we solve Eq. (11) and Eq. (12) and get four roots

$$\begin{aligned} x_1 &= \frac{-\sqrt{2+y} + \sqrt{2 - y - 2\sqrt{y^2 - 4(1-4\Delta)}}}{2}, \\ x_2 &= \frac{-\sqrt{2+y} - \sqrt{2 - y - 2\sqrt{y^2 - 4(1-4\Delta)}}}{2}, \\ x_3 &= \frac{\sqrt{2+y} + \sqrt{2 - y + 2\sqrt{y^2 - 4(1-4\Delta)}}}{2}, \\ x_4 &= \frac{\sqrt{2+y} - \sqrt{2 - y + 2\sqrt{y^2 - 4(1-4\Delta)}}}{2}. \end{aligned}$$

It can be verified that $|y| \leqslant 2$, so x_3 is the only root which is not less than 1 among $\{x_i \mid 1 \leqslant i \leqslant 4\}$, therefore

$$tr\sqrt{\rho} = \frac{\sqrt{2+y} + \sqrt{2 - y + 2\sqrt{y^2 - 4(1-4\Delta)}}}{2}.$$

This proof is finished.

3 Proof of Eq. (13) in the main text

We prove that $f \geqslant 0$ on $\mathcal{D}_f$, i.e. Eq. (13) in the main article holds. In order to find the stationary points for f in the interior of $\mathcal{D}_f$, take the partial derivative of f with respect to

the variables λ_{Δ_A} and λ_{det_A}, respectively, i.e.,

$$
\begin{aligned}
\frac{\partial f}{\partial \lambda_{\Delta_A}} &= f_{\lambda_{\Delta_A}}(\lambda_{A_1}, \lambda_{A_2}) \\
&= \frac{3u_4\left[u_3 - \frac{2(u_3+u_1)\left(u_6-\frac{2}{3}\right)-16}{u_5} + u_1\right]}{2u_2} - \frac{3\left(u_3+u_1\right)\left(u_6-\frac{2}{3}\right)-24}{u_5} - \frac{3\left(u_3+u_1\right)u_2}{2u_4}, \\
\frac{\partial f}{\partial \lambda_{det_A}} &= f_{\lambda_{det_A}}(\lambda_{A_1}, \lambda_{A_2}) \\
&= \frac{\sin(u_8)}{u_9\sqrt{-\left(\frac{p_A}{3}\right)^3}}\left\{\frac{32u_2u_7}{u_4} - \frac{3u_4}{2u_2}\left[\frac{64u_7}{3} - \frac{128\left(u_6-\frac{2}{3}\right)u_7}{3u_5}\right]\right. \\
&\qquad\left. + \frac{64\left(u_6-\frac{2}{3}\right)u_7}{u_5}\right\},
\end{aligned} \tag{15}
$$

where λ_{A_1} and λ_{A_2} are the eigenvalues of $\rho_A(|\psi\rangle_{ABC})$ and

$$
\begin{aligned}
u_1 &= \frac{2\sin(u_8)\left[\frac{32}{3\sqrt{-\left(\frac{p_A}{3}\right)^3}} + \frac{4\left(\frac{p_A}{3}\right)^2 q_A}{\left(-\frac{p_A}{3}\right)^{\frac{9}{2}}}\right]u_7}{3u_9}, \\
u_2 &= \sqrt{2u_5 - u_6 + \frac{8}{3}}, \\
u_3 &= \frac{16\cos(u_8)}{3u_7}, \\
u_4 &= \sqrt{u_6 + \frac{4}{3}}, \\
u_5 &= \sqrt{16\Delta_A - 4 + \left(u_6 - \frac{2}{3}\right)^2}, \\
u_6 &= 2\cos(u_8)u_7, \\
u_7 &= \sqrt{-\frac{p_A}{3}}, \\
u_8 &= \frac{\pi}{3} - \frac{1}{3}\arccos\left(\frac{q_A}{2\sqrt{-\left(\frac{p_A}{3}\right)^3}}\right), \\
u_9 &= \sqrt{\frac{q_A^2}{4\left(\frac{p_A}{3}\right)^3} + 1}.
\end{aligned}
$$

Consider the following equations

$$
\begin{cases}
f_{\lambda_{\Delta_A}}(\lambda_{A_1}, \lambda_{A_2}) = 0, \\
f_{\lambda_{det_A}}(\lambda_{A_1}, \lambda_{A_2}) = 0.
\end{cases} \tag{16}
$$

Through a complicated computation we find that Eqs. (16) has no solution, which implies that there is no stationary point for f in the interior of $\mathcal{D}_f$. Hence the minimum of f must be achieved on the boundaries of $\mathcal{D}_f$.

Next we check that $f \geqslant 0$ on the boundaries of $\mathcal{D}_f$. We shall break our proof up into the following cases based on the boundaries:

Case I $\partial_{\Delta_A=\frac{1}{3}}\mathcal{D}_f = \left\{\mathbf{X}_f \in \mathcal{D}_f | \Delta_A = \sum_{j=0}^{2}\left(\lambda_{j1}\lambda_{j2} + \lambda_{j1}\lambda_{j3} + \lambda_{j2}\lambda_{j3}\right) + \lambda_{\Delta_A} = \frac{1}{3}\right\}$,

Case II $\partial_{det\left[\rho_A(|\psi\rangle_{ABC})\right]=\frac{1}{27}}\mathcal{D}_f = \left\{\mathbf{X}_f \in \mathcal{D}_f | det\left[\rho_A(|\psi\rangle_{ABC})\right] = \sum_{j=0}^{2}\left(\lambda_{j1}\lambda_{j2}\lambda_{j3}\right) + \lambda_{det_A} = \frac{1}{27}\right\}$,

Case III $\partial_{\lambda_{ij}=0}\mathcal{D}_f = \{\mathbf{X}_f \in \mathcal{D}_f | \lambda_{ij} = 0\}$,

Case IV $\partial_{\lambda_{\Delta_A}=0}\mathcal{D}_f = \{\mathbf{X}_f \in \mathcal{D}_f | \lambda_{\Delta_A} = 0\}$,

Case V $\partial_{\lambda_{det_A}=0}\mathcal{D}_f = \{\mathbf{X}_f \in \mathcal{D}_f | \lambda_{det_A} = 0\}$.

3.1 Case I

We can prove that $\Delta_A = \lambda_{A_1}\lambda_{A_2} + \lambda_{A_1}\left(1 - \lambda_{A_1} - \lambda_{A_2}\right) + \lambda_{A_2}\left(1 - \lambda_{A_1} - \lambda_{A_2}\right) \leqslant \frac{1}{3}$ and the equality holds if and only if $\lambda_{A_1} = \lambda_{A_2} = \frac{1}{3}$. In this case, we have $\mathcal{E}^M_{A|BC} = 6$. By the result in Ref. [16] we know that $2\sum_{j=0}^{2} s_j \mathcal{E}^M(|\tau_j\rangle) \leqslant 4$. Thus $\mathcal{E}^M_{A|BC} > 2\sum_{j=0}^{2} s_j \mathcal{E}^M(|\tau_j\rangle)$ and Eq. (6) in the main text holds.

3.2 Case II

It can be proved that $det\left[\rho_A(|\psi\rangle_{ABC})\right] = \lambda_{A_1}\lambda_{A_2}\left(1 - \lambda_{A_1} - \lambda_{A_2}\right) \leqslant \frac{1}{27}$ and the equality holds if and only if $\lambda_{A_1} = \lambda_{A_2} = \frac{1}{3}$. Now the remaining part of proof follows from the corresponding discussion just as Case I.

3.3 Case III

In this case, a similar discussion just as Eqs. (16) implies that the munimum of f must be achieved on the boundaries of $\partial_{\lambda_{ij}=0}\mathcal{D}_f$. It follows that we only need to check the case: $\lambda_{i_0 j_0} = 1$ for any fixed i_0 and j_0, $\lambda_{ij} = 0$ for $i \neq i_0$ and $j \neq j_0$. In this case, it is obvious that $f \geqslant 0$.

3.4 Case IV

Denote

$$f_1 = f(\lambda_{01}, \lambda_{02}, \lambda_{03}, \lambda_{11}, \lambda_{12}, \lambda_{13}, \lambda_{21}, \lambda_{22}, 0, \lambda_{det_A}),$$

we need to prove that $f_1 \geq 0$. Let $\frac{\partial f_1}{\partial \lambda_{ij}} = 0$ with $i = 0, 1, 2$ and $j = 1, 2, 3$. It follows that λ_{ij} are all equal for all i, j. Note that $\sum_{i=0}^{2}\sum_{j=1}^{3}\lambda_{ij} = 1$, thus $\lambda_{ij} = \frac{1}{9}$. Substituting this and $\lambda_{\Delta_A} = 0$ into Eq. (15) we obtain that $\frac{\partial f}{\partial \lambda_{det_A}} > 60$. Therefore there is no stationary point for f_1 in the interior of $\partial_{\lambda_{\Delta_A}=0}\mathcal{D}_f$. Hence the minimum of f_1 must be achieved on the boundaries of $\partial_{\lambda_{\Delta_A}=0}\mathcal{D}_f$.

Next we show that $f_1 \geqslant 0$ on the boundaries of $\partial_{\lambda_{\Delta_A}=0}\mathcal{D}_f$. It is only needed to prove that the function

$$f_2 = f(\lambda_{01}, \lambda_{02}, \lambda_{03}, \lambda_{11}, \lambda_{12}, \lambda_{13}, \lambda_{21}, \lambda_{22}, 0, 0) \geqslant 0.$$

The analysis above tells us that the potential stationary point for f_2 is $\left(\frac{1}{9}, \frac{1}{9}, \frac{1}{9}, \frac{1}{9}, \frac{1}{9}, \frac{1}{9}, \frac{1}{9}, \frac{1}{9}, 0, 0\right)$.

Substituting this point into Eqs. (10) and (12) in the main text we get

$$\begin{cases} \lambda_{A_1}\lambda_{A_2} + \lambda_{A_1}(1-\lambda_{A_1}-\lambda_{A_2}) + \lambda_{A_2}(1-\lambda_{A_1}-\lambda_{A_2}) = \frac{1}{9}, \\ \lambda_{A_1}\lambda_{A_2}(1-\lambda_{A_1}-\lambda_{A_2}) = \frac{1}{243}. \end{cases} \tag{17}$$

Because Eqs. (17) has no real root, the point $(\frac{1}{9},\frac{1}{9},\frac{1}{9},\frac{1}{9},\frac{1}{9},\frac{1}{9},\frac{1}{9},\frac{1}{9},0,0)$ is not included in $\mathcal{D}_f$. Therefore there is no stationary point for f_2 in the interior of $\partial_{\lambda_{det_A}=0}\left(\partial_{\lambda_{\Delta_A}=0}\mathcal{D}_f\right)$. Thus the minimum of f_2 must be achieved on the boundaries of $\partial_{\lambda_{det_A}=0}\left(\partial_{\lambda_{\Delta_A}=0}\mathcal{D}_f\right)$ and the problem is reduced to Case III. Now the result follows from the discussion of Case III.

3.5 Case V

A similar discussion just as Case IV entails that

$$f_3 = f(\lambda_{01}, \lambda_{02}, \lambda_{03}, \lambda_{11}, \lambda_{12}, \lambda_{13}, \lambda_{21}, \lambda_{22}, \lambda_{\Delta_A}, 0) \geqslant 0.$$

The proof is completed.

4 Application

We will give a condition for the separability of a class of two-qutrit mixed states in a $3\otimes 3$ systems by an example. Take a mixed state ρ_{AB} in $3\otimes 3$ system $\mathcal{H}_A \otimes \mathcal{H}_B$ as follows.

$$\begin{aligned} \rho_{AB} = {} & t_1|0\rangle_A|0\rangle_{BA}\langle 0|_B\langle 0| + t_2|0\rangle_A|1\rangle_{BA}\langle 0|_B\langle 1| + t_3|0\rangle_A|2\rangle_{BA}\langle 0|_B\langle 2| \\ & + t_4|1\rangle_A|0\rangle_{BA}\langle 1|_B\langle 0| + t_5|1\rangle_A|1\rangle_{BA}\langle 1|_B\langle 1| + t_6|1\rangle_A|2\rangle_{BA}\langle 1|_B\langle 2| \\ & + t_7|2\rangle_A|0\rangle_{BA}\langle 2|_B\langle 0| + t_8|2\rangle_A|1\rangle_{BA}\langle 2|_B\langle 1| + t_9|2\rangle_A|2\rangle_{BA}\langle 2|_B\langle 2| \\ & + \sqrt{t_1t_4}\left(|1\rangle_A|0\rangle_{BA}\langle 0|_B\langle 0| + |0\rangle_A|0\rangle_{BA}\langle 1|_B\langle 0|\right) \\ & + \sqrt{t_2t_5}\left(|1\rangle_A|1\rangle_{BA}\langle 0|_B\langle 1| + |0\rangle_A|1\rangle_{BA}\langle 1|_B\langle 1|\right) \\ & + \sqrt{t_3t_6}\left(|1\rangle_A|2\rangle_{BA}\langle 0|_B\langle 2| + |0\rangle_A|2\rangle_{BA}\langle 1|_B\langle 2|\right) \\ & + \sqrt{t_4t_7}\left(|2\rangle_A|0\rangle_{BA}\langle 1|_B\langle 0| + |1\rangle_A|0\rangle_{BA}\langle 2|_B\langle 0|\right) \\ & + \sqrt{t_5t_8}\left(|2\rangle_A|1\rangle_{BA}\langle 1|_B\langle 1| + |1\rangle_A|1\rangle_{BA}\langle 2|_B\langle 1|\right) \\ & + \sqrt{t_6t_9}\left(|2\rangle_A|2\rangle_{BA}\langle 1|_B\langle 2| + |1\rangle_A|2\rangle_{BA}\langle 2|_B\langle 2|\right) \\ & + \sqrt{t_1t_7}\left(|2\rangle_A|0\rangle_{BA}\langle 0|_B\langle 0| + |0\rangle_A|0\rangle_{BA}\langle 2|_B\langle 0|\right) \\ & + \sqrt{t_2t_8}\left(|2\rangle_A|1\rangle_{BA}\langle 0|_B\langle 1| + |0\rangle_A|1\rangle_{BA}\langle 2|_B\langle 1|\right) \\ & + \sqrt{t_3t_9}\left(|2\rangle_A|2\rangle_{BA}\langle 0|_B\langle 2| + |0\rangle_A|2\rangle_{BA}\langle 2|_B\langle 2|\right), \end{aligned}$$

where $t_i \geqslant 0$ $(1 \leqslant t \leqslant 9)$, $\sum_{i=1}^{9} t_i = 1$, $t_1t_5 = t_2t_4$ and $t_1t_6 = t_3t_4$. It can be symmetrically extended into a pure state $|\psi\rangle_{ABC}$ in $3\otimes 3\otimes 3$ system $\mathcal{H}_A \otimes \mathcal{H}_B \otimes \mathcal{H}_C$,

$$\begin{aligned} |\psi\rangle_{ABC} = {} & \sqrt{t_1}|000\rangle + \sqrt{t_2}|011\rangle + \sqrt{t_3}|022\rangle + \sqrt{t_4}|100\rangle + \sqrt{t_5}|111\rangle + \sqrt{t_6}|122\rangle \\ & + \sqrt{t_7}|200\rangle + \sqrt{t_8}|211\rangle + \sqrt{t_9}|222\rangle. \end{aligned}$$

One can easily check that $\rho_{AB} = tr_C\left(|\psi\rangle_{ABC}\right)$ and $\rho_{AB} = \rho_{AC}$. It follows from the monogamous relation $\mathcal{E}^M_{AB} + \mathcal{E}^M_{AC} \leqslant \mathcal{E}^M_{A|BC}$ that $\mathcal{E}^M_{AB} \leqslant \frac{1}{2}\mathcal{E}^M_{A|BC}$.

Next we are going to find out the condition for the separability of ρ_{AB}. Let

$$\begin{aligned}
t_1 &= a_1a_2a_3,\\
t_2 &= a_1a_2a_4,\\
t_3 &= a_1a_2(1-a_3-a_4),\\
t_4 &= a_1a_3(1-a_2),\\
t_5 &= a_1a_4(1-a_2),\\
t_6 &= a_1(1-a_2)(1-a_3-a_4),\\
t_7 &= (1-a_1)a_5,\\
t_8 &= (1-a_1)a_6,\\
t_9 &= (1-a_1)(1-a_5-a_6),
\end{aligned}$$

where $a_j \geqslant 0$ $(1 \leqslant j \leqslant 6)$. Then,

1. when $a_1 = 1$, i.e., $t_7 = t_8 = t_9 = 0$, we obtain

$$|\psi\rangle_{ABC} = \left(\sqrt{a_2}|0\rangle_A + \sqrt{1-a_2}|1\rangle_A\right)\otimes\left(\sqrt{a_3}|0\rangle_B|0\rangle_C + \sqrt{a_4}|1\rangle_B|1\rangle_C + \sqrt{1-a_3-a_4}|2\rangle_B|2\rangle_C\right),$$

which is separable. Hence $\mathcal{E}^M_{AB} \leqslant \frac{1}{2}\mathcal{E}^M_{A|BC} = 0$, namely, ρ_{AB} is separable;

2. when $a_1 = 0$, i.e., $t_i = 0$ $(1 \leqslant i \leqslant 6)$, we obtain

$$|\psi\rangle_{ABC} = |2\rangle_A \otimes \left(\sqrt{a_5}|0\rangle_B|0\rangle_C + \sqrt{a_6}|1\rangle_B|1\rangle_C + \sqrt{1-a_5-a_6}|2\rangle_B|2\rangle_C\right),$$

which is separable. Hence $\mathcal{E}^M_{AB} \leqslant \frac{1}{2}\mathcal{E}^M_{A|BC} = 0$ and ρ_{AB} is separable;

3. when $a_1 = \frac{1}{2}$, $a_3 = a_5$ and $a_4 = a_6$, i.e., $t_7 + t_8 + t_9 = \frac{1}{2}$, we obtain

$$\begin{aligned}
|\psi\rangle_{ABC} = \sqrt{\frac{1}{2}}\left(\sqrt{a_2}|0\rangle_A + \sqrt{1-a_2}|1\rangle_A + |2\rangle_A\right) \otimes \left(\sqrt{a_3}|0\rangle_B|0\rangle_C + \sqrt{a_4}|1\rangle_B|1\rangle_C\right.\\
\left. + \sqrt{1-a_3-a_4}|2\rangle_B|2\rangle_C\right),
\end{aligned}$$

which is separable. Hence $\mathcal{E}^M_{AB} \leqslant \frac{1}{2}\mathcal{E}^M_{A|BC} = 0$ and ρ_{AB} is separable.

Therefore, ρ_{AB} is separable when $t_7 = t_8 = t_9 = 0$ or $t_i = 0$ $(1 \leqslant i \leqslant 6)$ or $t_7 + t_8 + t_9 = \frac{1}{2}$.
